# Supplementary material for: The Transcription Factor STAT6 Mediates Direct Repression of Inflammatory Enhancers and Limits Activation of Alternatively Polarized Macrophages
Source: Immunity. 2018 Jan 16;48(1):75–90.e6. doi: 10.1016/j.immuni.2017.12.010 (PMC5772169; doi:10.1016/j.immuni.2017.12.010)
Supplement: Document S1. Figures S1–S7 [file mmc1.pdf]

Immunity, Volume *48*

Supplemental Information

The Transcription Factor STAT6 Mediates Direct Repression of Inflammatory Enhancers and Limits Activation of Alternatively Polarized Macrophages

Zsolt Czimmerer, Bence Daniel, Attila Horvath, Dominik Rückerl, Gergely Nagy, Mate Kiss, Matthew Peloquin, Marietta M. Budai, Ixchelt Cuaranta-Monroy, Zoltan Simandi, Laszlo Steiner, Bela Nagy Jr., Szilard Poliska, Csaba Banko, Zsolt Bacso, Ira G. Schulman, Sascha Sauer, Jean-Francois Deleuze, Judith E. Allen, Szilvia Benko, and Laszlo Nagy

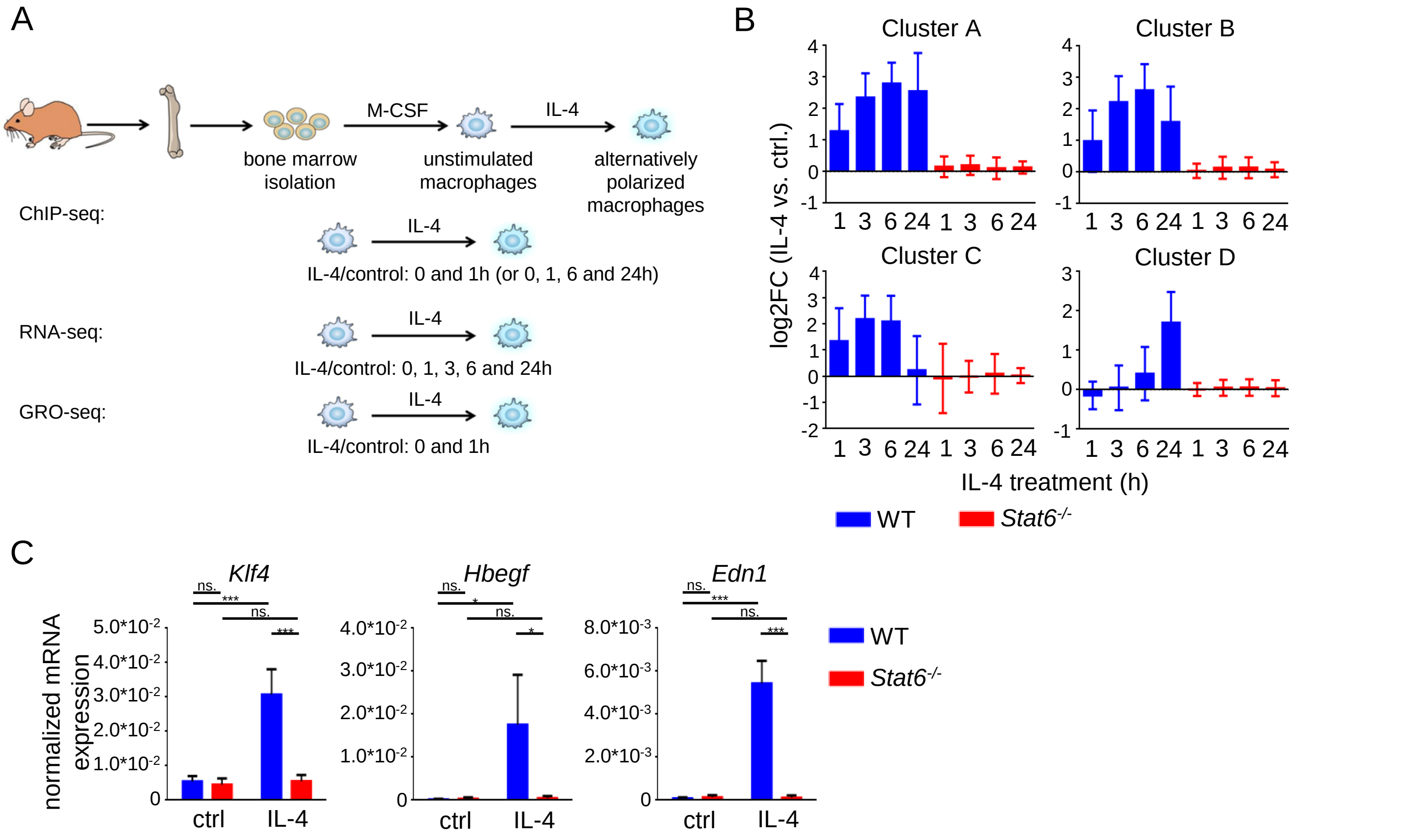

Figure S1. (Related to Figure 1) Identification of IL-4-STAT6 signaling pathway-mediated global transcriptional changes in mouse BMDMs. (A) Schematic representation of applied mouse alternative macrophage polarization protocol and experimental system. (B) The average fold change from the IL-4-repressed gene cluster at the indicated time points following IL-4 stimulation in WT (n=4) and *Stat6*$^{-/-}$ (n=2) BMDMs. Error bars represent means ±SD. (C) RT-qPCR analysis of gene expression on a set of IL-4-activated genes in WT and Stat6$^{-/-}$ BMDMs. BMDMs were treated with IL-4 for 6 hours. Data are representative of five individual animals per genotype from two independent experiments. *P<0.05, **P<0.01, ***P<0.001, ns. indicates not significant change. Error bars represent means ±SD.

A

	RNAPII-pS2⁺ gene bodies
All	18034
IL-4-repressed	3008
IL-4-induced	2923

B

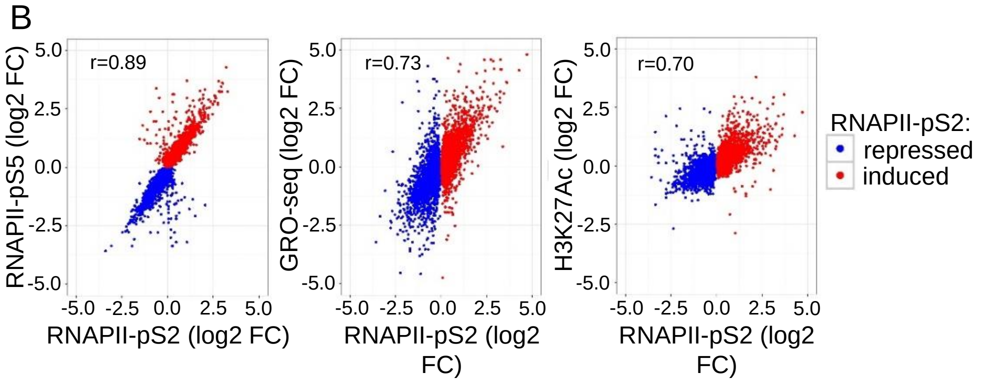

C

RNAPII-pS2 positive gene bodies

Gene expression clusters (Figure 1A)	IL-4-repressed	IL-4-induced
A (32)	0	31
B (164)	1	142
C (292)	3	235
D (501)	125	137
E (625)	406	18

D

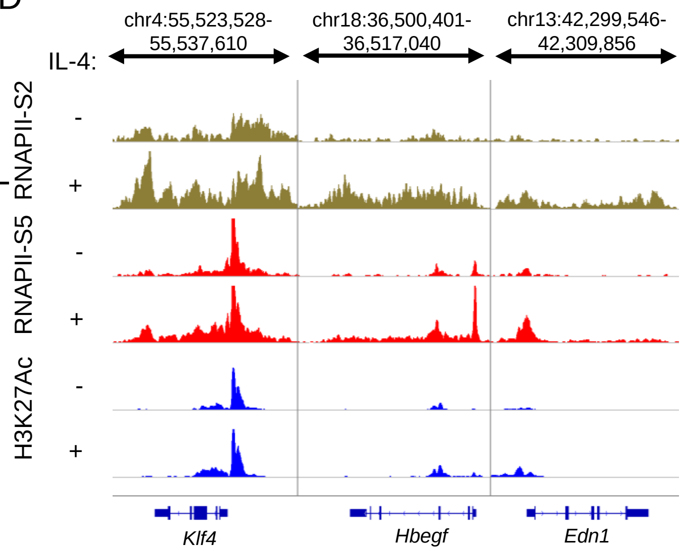

Figure S2. (Related to Figure 2) Characterization of RNAPII binding, nascent RNA expression and H3K27Ac enrichment at the gene bodies of IL-4-responsive genes. (A) The number of gene bodies associated with IL-4-dependent regulation of RNAPII-pS2 binding (p≤0.1) in BMDMs. (B) Correlation of RNAPII-pS2 binding with RNAPII-pS5 binding, nascent RNA expression as well as H3K27Ac enrichment at the IL-4-regulated RNAPII-pS2 binding-associated gene bodies. Data (H3K27Ac, RNAPII-pS2 and RNAPII-pS5) are combined from two independent biological replicates. (C) Overlap between IL-4-regulated gene expression clusters (Figure 1A) and IL-4-regulated RNAPII-pS2-associated gene bodies (Figure S2A). (D) H3K27Ac, RNAPII-pS5 and RNAPII-pS2 ChIP-Seq signals at the selected IL-4-activated gene bodies. ChIP-seq signals are visualized by the Integrative Genomics Viewer. Data are representative of two independent biological replicates.

BMDMs were treated with IL-4 for 1 hour in (A, B and D).

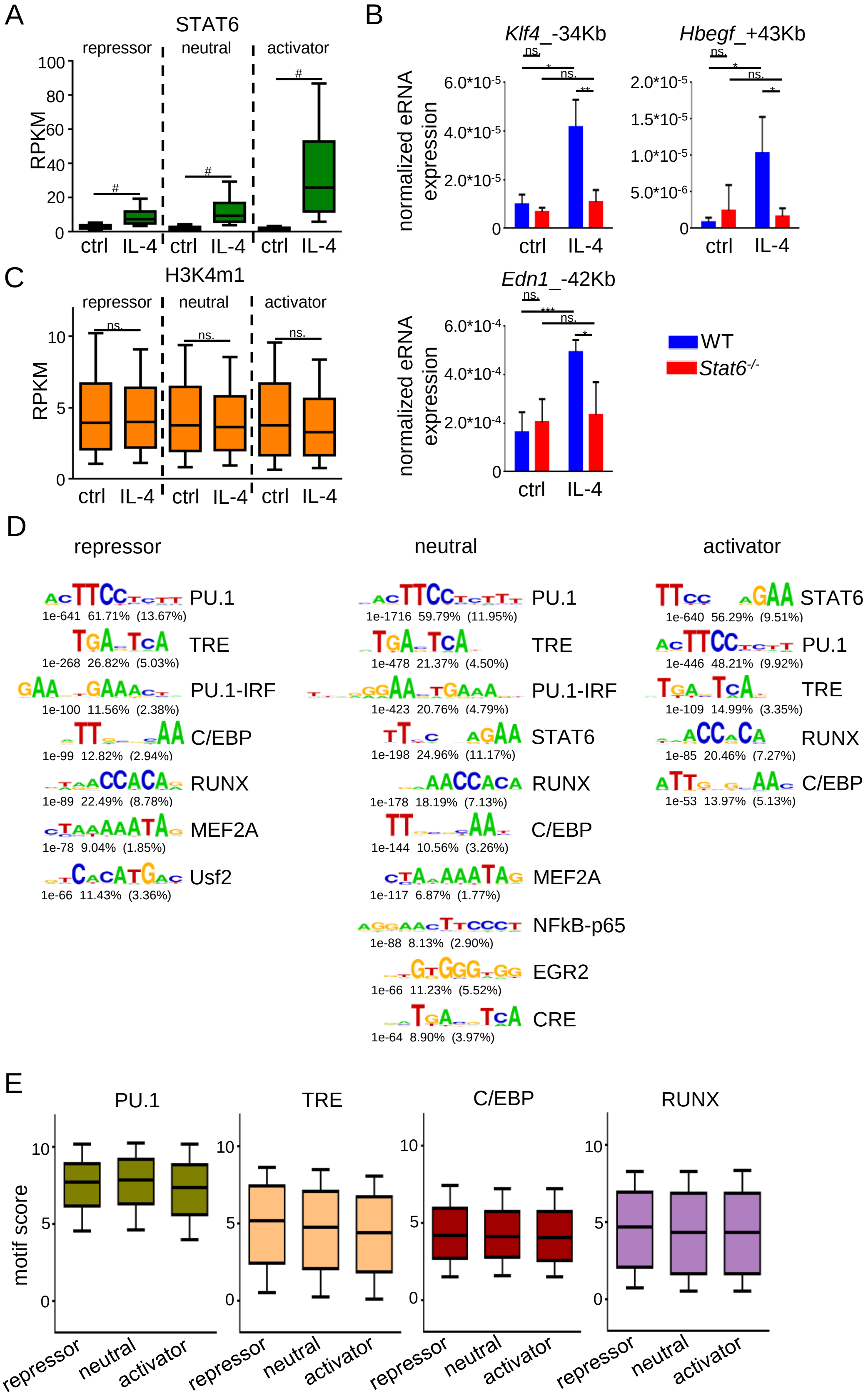
A
STAT6
repressor
neutral
activator
RPKM
ctrl
IL-4
B
Klf4_-34Kb
Hbegf_+43Kb
normalized eRNA expression
C
H3K4m1
Edn1_-42Kb
WT
Stat6-/-
D
repressor
neutral
activator
PU.1
TRE
PU.1-IRF
C/EBP
RUNX
MEF2A
Usf2
STAT6
NFkB-p65
EGR2
CRE
E
PU.1
TRE
C/EBP
RUNX
motif score

Figure S3. (Related to Figure 3) Characterization of RNAPII-pS5 positive STAT6 peak clusters. (A) Box plot representation of STAT6 transcription factor binding at the STAT6-occupied RNAPII-pS5^{+} genomic regions in WT macrophages. BMDMs were treated with IL-4 for 1 hour. Boxes encompass the 25th to 75th percentile RPKMs. Whiskers extend to the 10th and 90th percentiles. Changes were considered significant at $p<0.00001$ using paired t-test and the average of fold differences at the individual enhancers ≥1.15. # $p<0.00001$ and average fold difference≥1.15, ns. indicates not significant. (B) RT-qPCR measurements of eRNA expression at IL-4-repressed enhancers in WT and *Stat6*$^{-/-}$ macrophages. BMDMs were treated with IL-4 for 1 hour. Data are representative of five individual animals per genotype from two independent experiments. *$P<0.05$, **$P<0.01$, ***$P<0.001$, ns. indicates not significant change. Error bars represent means ±SD. (C) Box plot representation of H3K4m1 enrichment at the surrounding genomic regions of the identified STAT6 peak clusters in WT macrophages. Macrophages were treated with IL-4 for 4 hours. Boxes encompass the 25th to 75th percentile RPKMs. Whiskers extend to the 10th and 90th percentiles. Changes were considered significant at $p<0.00001$ using paired t-test and the average of fold differences at the individual enhancers≥1.15. # $p<0.00001$ and average fold difference≥1.15, ns. indicates not significant. (D) De novo motif enrichment identification under different STAT6 peak clusters from ChIP-seq data using HOMER. “Target %” refers to the ratio of the peaks having the given motif, and “Bg %” refers to the ratio of a random background. (E) Box plot representation of PU.1, TRE, C/EBP and RUNX motif scores on genomic regions under different STAT6 peak clusters. Boxes encompass the 25th to 75th percentile motif scores. Whiskers extend to the 10th and 90th percentiles.

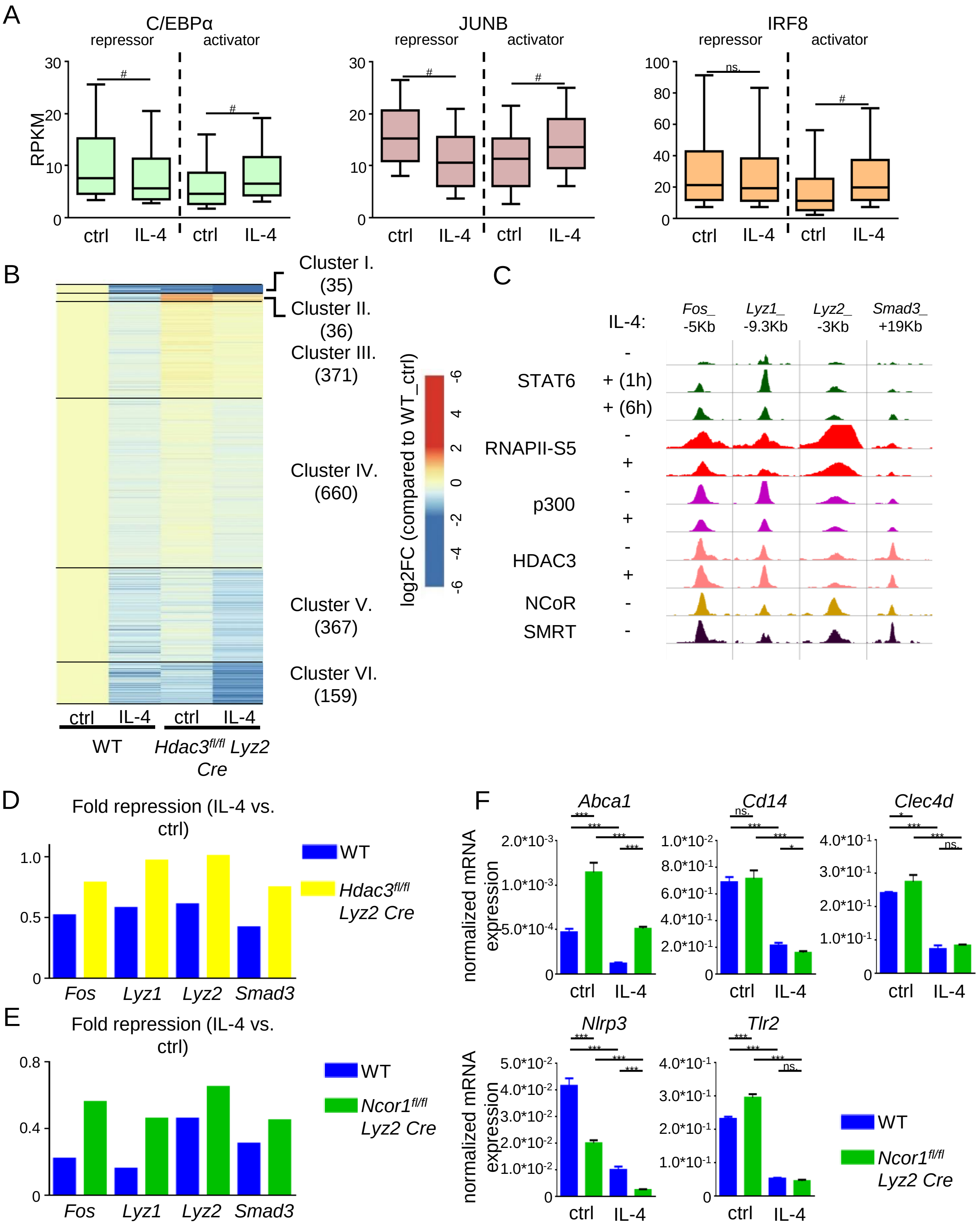

A
C/EBPα
JUNB
IRF8
repressor
activator
RPKM
ctrl
IL-4
B
Cluster I. (35)
Cluster II. (36)
Cluster III. (371)
Cluster IV. (660)
Cluster V. (367)
Cluster VI. (159)
log2FC (compared to WT_ctrl)
WT
Hdac3fl/fl Lyz2 Cre
C
IL-4:
Fos_ -5Kb
Lyz1_ -9.3Kb
Lyz2_ -3Kb
Smad3_ +19Kb
STAT6
+ (1h)
+ (6h)
RNAPII-S5
p300
HDAC3
NCoR
SMRT
D
Fold repression (IL-4 vs. ctrl)
WT
Hdac3fl/fl Lyz2 Cre
Fos
Lyz1
Lyz2
Smad3
E
Fold repression (IL-4 vs. ctrl)
WT
Ncor1fl/fl Lyz2 Cre
F
Abca1
Cd14
Clec4d
Nlrp3
Tlr2
normalized mRNA expression
WT
Ncor1fl/fl Lyz2 Cre

Figure S4. (Related to Figure 4) Characterization of IL-4-STAT6 signaling pathway-mediated transcriptional re-ression and activation at the STAT6 bound enhancers. (A) Box plot representation of JunB, C/EBPα and IRF8 ChIP-seq signals at the STAT6-occupied genomic regions in WT macrophages. BMDMs were treated with IL-4 for 1 hour. Boxes encompass the 25th to 75th percentile RPKMs. Whiskers extend to the 10th and 90th percentiles. Changes were considered significant at $p<0.00001$ using paired t-test and the average of fold differences at the individual enhancers≥1.15. # $p<0.00001$ and average fold difference≥1.15, ns. indicates not significant. (B) Heat map representation of IL-4-repressed gene clusters ($p \le 0.05$) in unstimulated and IL-4 stimulated WT and *Hdac3$^{fl/fl}$ Lyz2 Cre* murine macrophages. Clustering was based on the participation of HDAC3 in the IL-4-mediated repression. BMDMs were treated with IL-4 for 24 hours. (C) Representative examples of IL-4-STAT6-HDAC3-repressed genes-associated enhancers. Genome browser view of the merge of STAT6, RNAPII-pS5, p300 and HDAC3-specific ChIP-seq from unstimulated or IL-4-stimulated WT as well as SMRT and NCoR ChIP-seq from unstimulated WT macrophages. (D) Fold repression of *Fos*, *Lyz1*, *Lyz2* and *Smad3* expression between IL-4-stumulated and unstimulated WT as well as *Hdac3$^{fl/fl}$ Lyz2 Cre* BMDMs. BMDMs were treated with IL-4 for 24 hours. (E) Fold repression of *Fos*, *Lyz1*, *Lyz2* and *Smad3* expression between IL-4-stumulated and unstimulated WT as well as *Ncor1$^{fl/fl}$ Lyz2 Cre* iBMDMs. BMDMs were treated with IL-4 for 24 hours. (F) RT-qPCR-based measurement of *Abca1*, *Cd14*, *Clec4d*, *Nlrp3* and *Tlr2* expression in unstimulated as well as IL-4 stimulated WT and *Ncor1$^{fl/f}$ Lyz2 Cre* iBMDMs. BMDMs were treated with IL-4 for 24 hours. Data represent the mean and SD of three independent biological replicates. *$P<0.05$, **$P<0.01$, ***$P<0.001$, ns. indicates no significant difference.

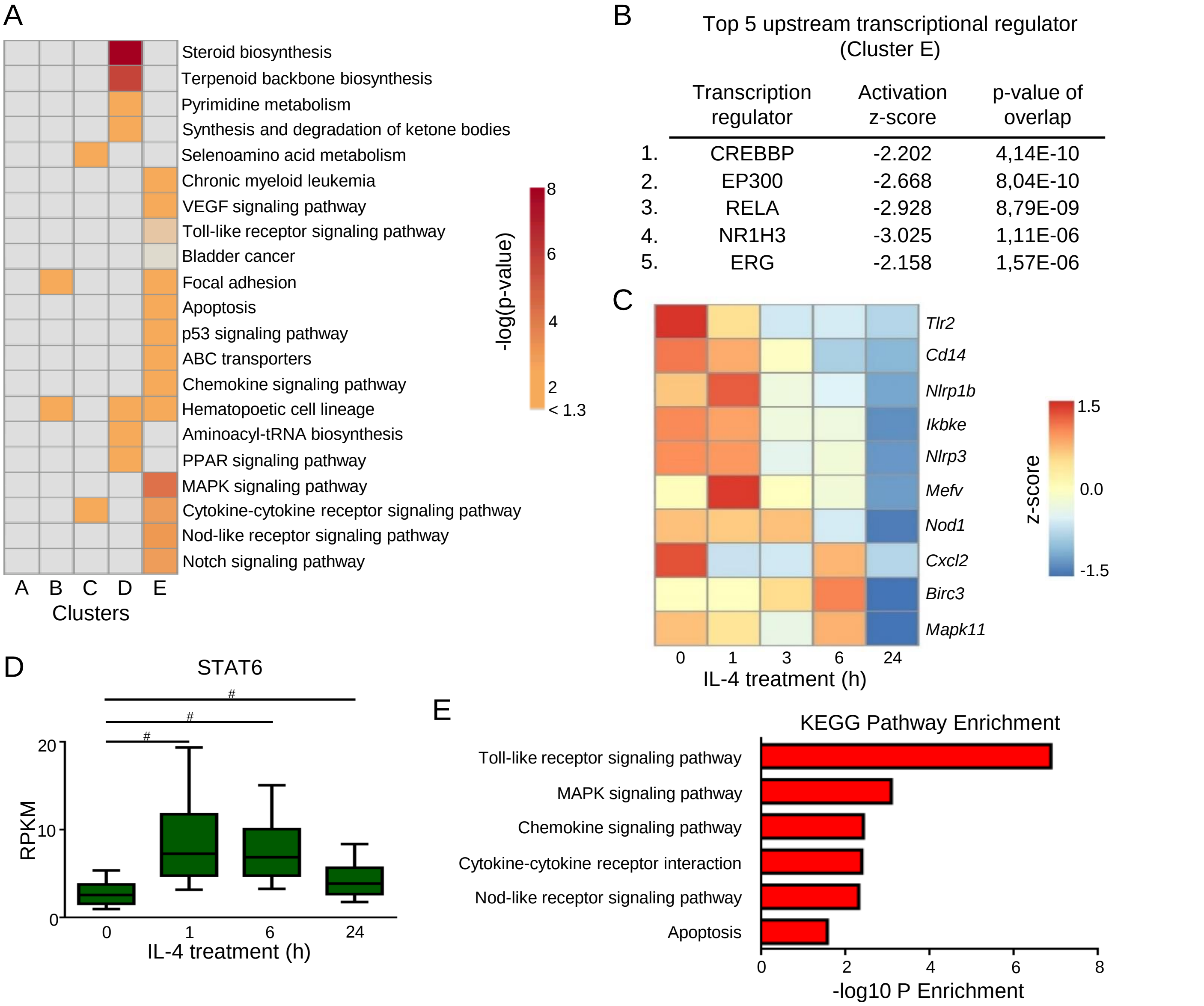

Figure S5. (Related to Figure 5) (A) Five IL-4-regulated gene clusters-associated significantly enriched KEGG pathway categories. (B) Ingenuity Pathway Analysis algorithm-based prediction of top upstream transcriptional regulators of IL-4-repressed genes. Transcription regulators showing *p*-value overlap <0.01 and regulation *z*-score >2 or <-2 are shown. (C) Heat map representation of IL-4-regulated gene expression of the selected members of Toll-like and Nod-like receptor pathways in murine BMDMs. BMDMs were treated with IL-4 for 1, 3, 6 and 24 hours. (D) Box plot representation of STAT6 transcription factor binding at the "repressor" STAT6-occupied RNAPII-pS5^{+} genomic regions in WT BMDMs. BMDMs were treated with IL-4 for 1, 6 and 24 hours. Boxes encompass the 25th to 75th percentile RPKMs. Whiskers extend to the 10th and 90th percentiles. Changes were considered significant at p<0.00001 using paired t-test and the average of fold differences at the individual enhancers ≥1.15. # p<0.00001 and average fold difference≥1.15, ns. indicates not significant. (E) IL-4-attenuated LPS-inducible gene cluster-associated significantly enriched KEGG pathway categories.

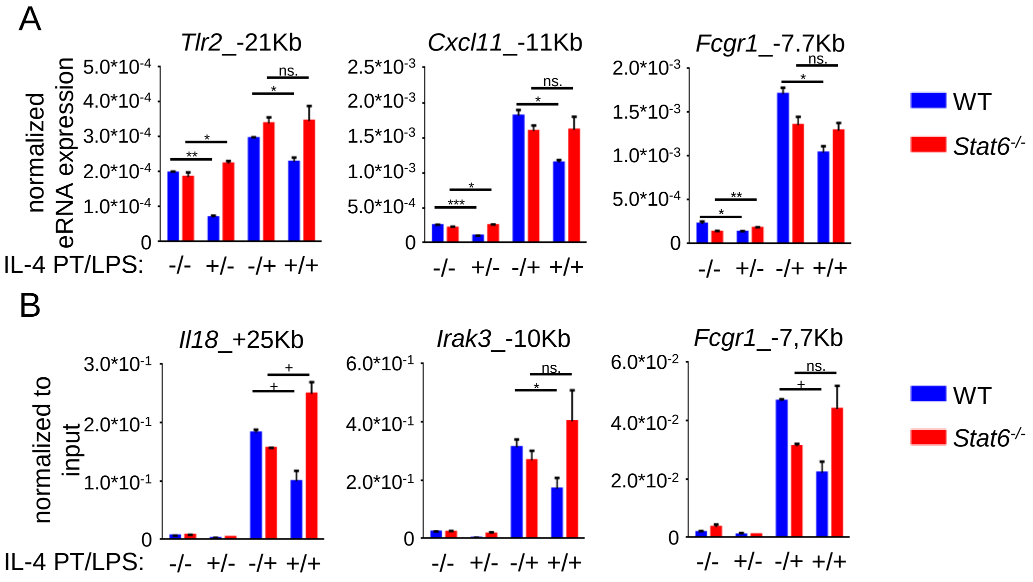

Figure S6. (Related to Figure 6) Investigation of IL-4 pretreatment-madiated repression at the selected LPS-activated enhancers. (A) RT-qPCR-based measurement of basal and LPS-induced eRNA expression at the selected enhancers in IL-4-pre-treated and unstimulated WT and *Stat6*$^{-/-}$ iBMDMs. iBMDMs were pretreated with IL-4 for 24 hours followed by 3 hours LPS exposure. Data represent the mean and SD of three biological replicates. **P*<0.05, ***P*<0.01, ****P*<0.001, ns. indicates not significant. (B) ChIP-qPCR-based measurement of p65 binding at the selected IL-4-repressed LPS-responsive enhancers from WT and *Stat6*$^{-/-}$ BMDMs. BMDMs were pretreated with IL-4 for 24 hours followed by 1 hour LPS exposure. Data represent the mean and SD of two biological replicates. $^{+}$*P*<0.1, **P*<0.5, ns. indicates not significant.

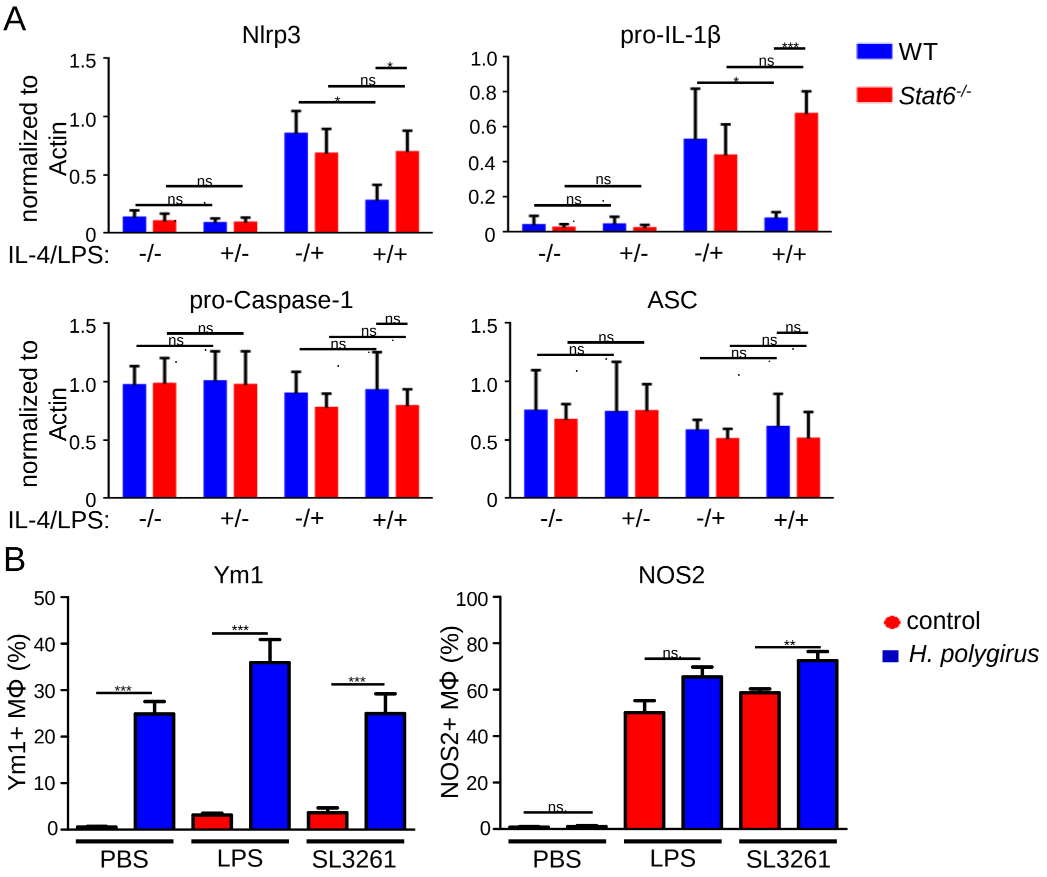

Figure S7. (Related to Figure 7) (A) Western blot densitometry analysis of basal and LPS-regulated Nlrp3, pro-IL-1β, pro-Caspase1, ASC and β-actin expression at protein levels in IL-4-pre-treated and unstimulated WT and *Stat6*$^{-/-}$ mouse BMDMs. BMDMs were pretreated with IL-4 for 24 hours followed by 3 hours LPS exposure. Data represent the mean and SD of five individual animals from two independent experiments. **P*<0.05, ***P*<0.01, ****P*<0.001, ns. indicates not significant. (B) The percentage of Ym1 positive and NOS2 positive peritoneal macrophage number in control and *Heligmosomoides polygyrus* (H. polygyrus)-infected mice following PBS and LPS injection as well as *Salmonella Typhimurium* (SL3261)-infection. Data represent the mean and SD of four-eleven individual animals. **P*<0.05, ***P*<0.01, ****P*<0.001, ns. indicates not significant.
