# Supplementary material for: The Transcription Factor STAT6 Mediates Direct Repression of Inflammatory Enhancers and Limits Activation of Alternatively Polarized Macrophages
Source: Immunity. 2018 Jan 16;48(1):75–90.e6. doi: 10.1016/j.immuni.2017.12.010 (PMC5772169; doi:10.1016/j.immuni.2017.12.010)
Supplement: Document S2. Article plus Supplemental Information [file mmc9.pdf]

Immunity

Article

The Transcription Factor STAT6 Mediates Direct Repression of Inflammatory Enhancers and Limits Activation of Alternatively Polarized Macrophages

Graphical Abstract

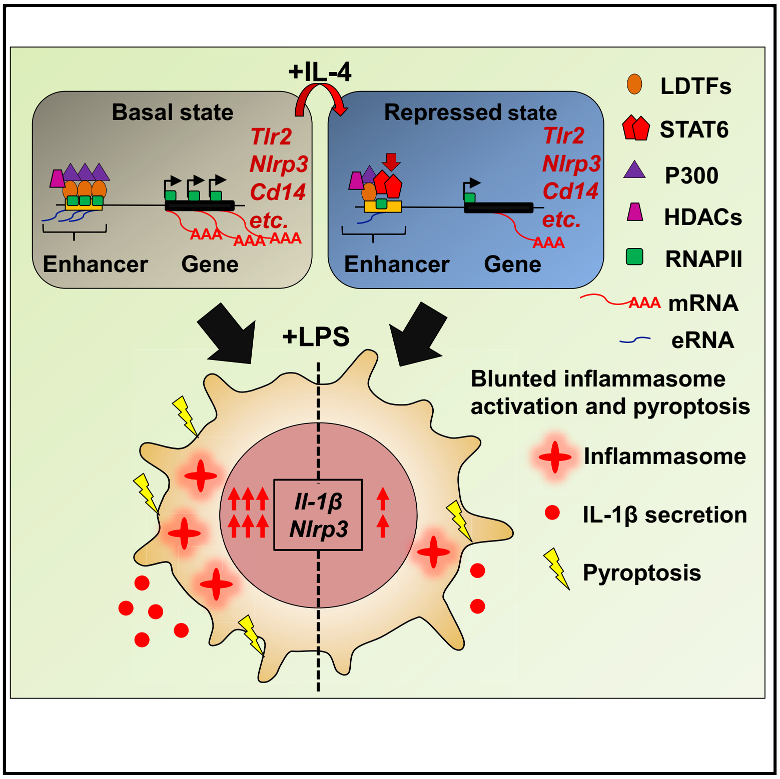

Highlights

- IL-4-activated STAT6 acts as a transcriptional repressor in macrophages
- IL-4-STAT6-repressed enhancers associate with reduced LDTF and p300 binding
- Inflammatory responsiveness of the IL-4-repressed enhancers is attenuated
- IL-4 limits the LPS-induced inflammasome activation, IL-1β production, and pyroptosis

Authors

Zsolt Czimmerer, Bence Daniel, Attila Horvath, ..., Judith E. Allen, Szilvia Benko, Laszlo Nagy

Correspondence

lnagy@sbpdiscovery.org

In Brief

The molecular bases of repressive transcriptional mechanisms contributing to macrophage polarization are not well understood. Czimmerer et al. show that in alternatively polarized macrophages, IL-4-activated STAT6 represses a large set of enhancers modulating the transcriptional program. STAT6-repressed enhancers are characterized by reduced chromatin accessibility, eRNA expression, LDTF, and p300 binding. IL-4-STAT6-mediated repression limits the inflammatory responsiveness including inflammasome activation, IL-1β production, and pyroptosis. Thus, the IL4-STAT6 pathway establishes an epigenomic signature to selectively repress the macrophage inflammation program.

Czimmerer et al., 2018, Immunity *48*, 75–90
January 16, 2018 © 2017 The Authors. Published by Elsevier Inc.
https://doi.org/10.1016/j.immuni.2017.12.010

CellPress

CellPress

The Transcription Factor STAT6 Mediates Direct Repression of Inflammatory Enhancers and Limits Activation of Alternatively Polarized Macrophages

Zsolt Czimmerer,[1,15] Bence Daniel,[2,15] Attila Horvath,[1,15] Dominik Rückerl,[3,15] Gergely Nagy,[1,14] Mate Kiss,[1] Matthew Peloquin,[2] Marietta M. Budai,[4] Ixchelt Cuaranta-Monroy,[1] Zoltan Simandi,[2] Laszlo Steiner,[5] Bela Nagy, Jr.,[6] Szilard Poliska,[7] Csaba Banko,[8] Zsolt Bacso,[8] Ira G. Schulman,[9] Sascha Sauer,[10,11,12] Jean-Francois Deleuze,[13] Judith E. Allen,[3] Szilvia Benko,[4] and Laszlo Nagy[1,2,14,16,*]

[1]Department of Biochemistry and Molecular Biology, Faculty of Medicine, University of Debrecen, Debrecen, Hungary
[2]Sanford-Burnham-Prebys Medical Discovery Institute, 6400 Sanger Road, Orlando, FL 32827, USA
[3]Faculty of Biology, Medicine and Health, School of Biological Sciences, University of Manchester, Manchester, UK
[4]Department of Physiology, Faculty of Medicine, University of Debrecen, Debrecen, Hungary
[5]UD-Genomed Medical Genomic Technologies Ltd., Debrecen, Hungary
[6]Department of Laboratory Medicine, Faculty of Medicine, University of Debrecen, Debrecen, Hungary
[7]Genomic Medicine and Bioinformatic Core Facility, Department of Biochemistry and Molecular Biology, Faculty of Medicine, University of Debrecen, Debrecen, Hungary
[8]Department of Biophysics and Cell Biology, Faculty of Medicine, University of Debrecen, Debrecen, Hungary
[9]Department of Pharmacology, University of Virginia, Charlottesville, VA, USA
[10]Otto Warburg Laboratory, Max Planck Institute for Molecular Genetics, Berlin, Germany
[11]CU Systems Medicine, University of Würzburg, Würzburg, Germany
[12]Max Delbrück Center for Molecular Medicine (BIMSB and BIH), Berlin, Germany
[13]Centre National de Génotypage, Institut de Génomique, CEA, Evry, France
[14]MTA-DE "Lendület" Immunogenomics Research Group, University of Debrecen, Debrecen, Hungary
[15]These authors contributed equally
[16]Lead Contact
*Correspondence: lnagy@sbpdiscovery.org
https://doi.org/10.1016/j.immuni.2017.12.010

SUMMARY

The molecular basis of signal-dependent transcriptional activation has been extensively studied in macrophage polarization, but our understanding remains limited regarding the molecular determinants of repression. Here we show that IL-4-activated STAT6 transcription factor is required for the direct transcriptional repression of a large number of genes during *in vitro* and *in vivo* alternative macrophage polarization. Repression results in decreased lineage-determining transcription factor, p300, and RNA polymerase II binding followed by reduced enhancer RNA expression, H3K27 acetylation, and chromatin accessibility. The repressor function of STAT6 is HDAC3 dependent on a subset of IL-4-repressed genes. In addition, STAT6-repressed enhancers show extensive overlap with the NF-κB p65 cistrome and exhibit decreased responsiveness to lipopolysaccharide after IL-4 stimulus on a subset of genes. As a consequence, macrophages exhibit diminished inflammasome activation, decreased IL-1β production, and pyroptosis. Thus, the IL-4-STAT6 signaling pathway establishes an alternative polarization-specific epigenomic signature resulting in dampened macrophage responsiveness to inflammatory stimuli.

INTRODUCTION

Macrophage plasticity is ensured by dynamic and partially reversible responsiveness to pathogen-derived molecules as well as the cytokine and lipid microenvironment. The two well-characterized extreme functional outcomes of macrophage polarization are T helper 1 (Th1) cell-type cytokine interferon-gamma (IFN-γ)-induced classical or M(INF-γ)-type polarization with enhanced bactericidal capacity and Th2 cell-type cytokine interleukin-4 (IL-4)-induced alternative or M(IL-4)-type polarization with anti-inflammatory properties, but complex molecular cues can generate an entire spectrum of different activation states (Gordon and Martinez, 2010; Murray et al., 2014).

The major determinant of macrophage plasticity is their specific transcriptional program dictated primarily by lineage-determining transcription factors (LDTFs) including ETS-domain transcription factor PU.1, CCAAT/enhancer binding proteins (C/EBPs), activator protein 1 (AP-1), or Runt-related transcription factor 1 (RUNX1) as well as extracellular signal-dependent transcription factors (SDTFs) including LPS-activated nuclear factor kappa-light-chain-enhancer of activated B cells (NF-κB) or AP-1, IFN-γ-activated signal transducer and activator of transcription 1 (STAT1), or IL-4- and IL-13-activated STAT6; for a review see Glass and Natoli (2016). Despite the fact that polarization signals repress large sets of genes, the repressive activity of polarization-specific transcription factors has not been studied in detail (Bhatt et al., 2012; Martinez et al., 2013). Recently, a whole new spectrum of next-generation sequencing-based methods has evolved, enabling the characterization of the molecular

This is an open access article under the CC BY license (http://creativecommons.org/licenses/by/4.0/).

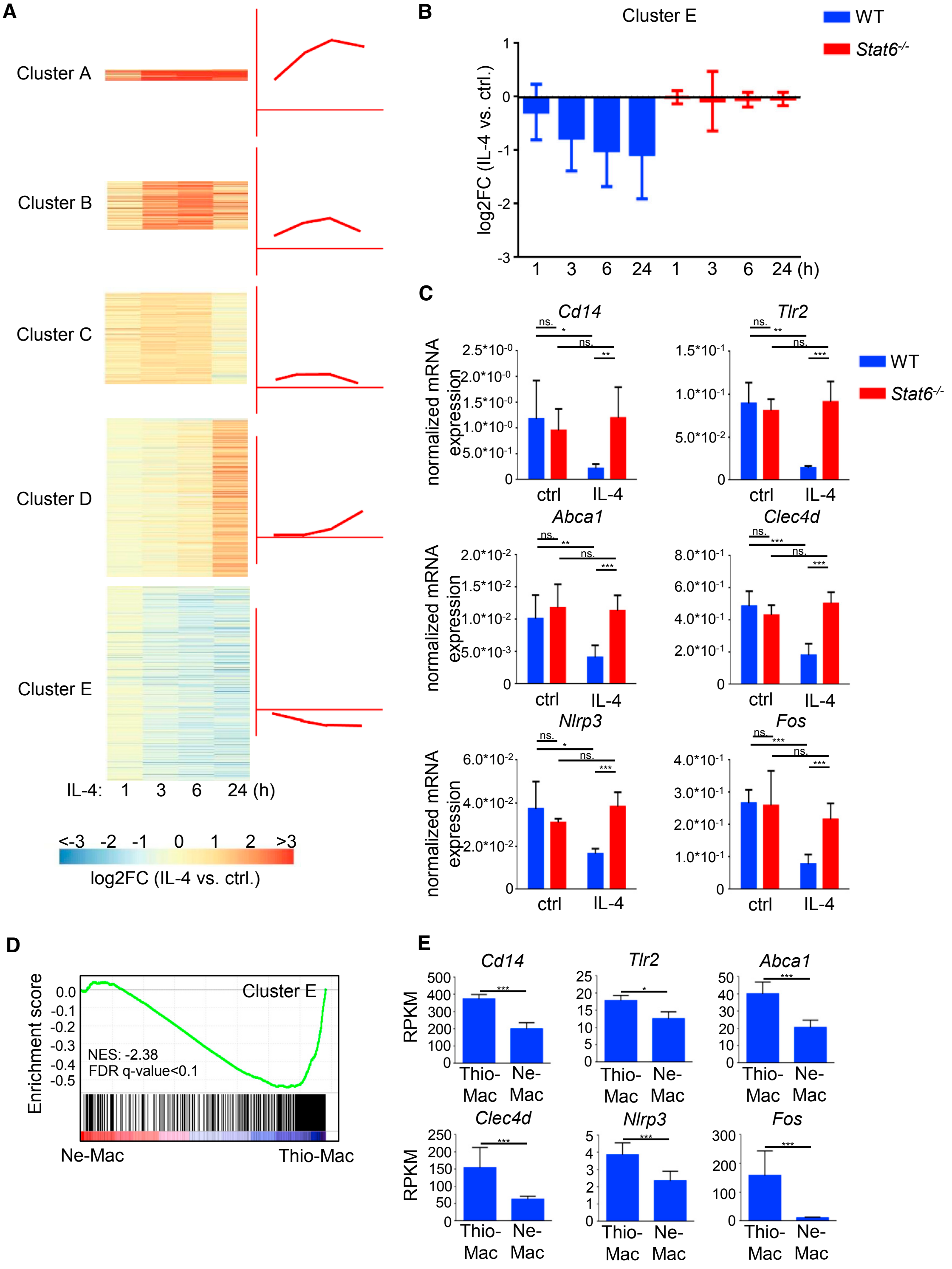

Figure 1. The IL-4-STAT6 Signaling Pathway Induced Gene Expression during Mouse Alternative Macrophage Polarization

(A) Heatmap of IL-4-regulated (p value $\leq$ 0.05, FC $\geq$ 2) gene expression clusters in WT murine BMDMs. Data represent the average fold changes of four individual animals.

(B) The average fold change from the IL-4-repressed gene cluster at the indicated time points following IL-4 stimulation in WT (n = 4) and *Stat6*$^{-/-}$ (n = 2) BMDMs. Error bars represent means ± SD.

(C) RT-qPCR analysis of gene expression on a set of IL-4-repressed genes in WT and *Stat6*$^{-/-}$ BMDMs. BMDMs were treated with IL-4 for 6 hr. Data are representative of five individual animals per genotype from two independent experiments. *p < 0.05, **p < 0.01, ***p < 0.001, ns, not significant change. Error bars represent means ± SD.

(legend continued on next page)

features of transcriptional repression in macrophages at an unprecedented level (Hah et al., 2015; Kang et al., 2017).

IL-4- or IL-13-induced alternative macrophage polarization occurs in a number of pathological processes including nematode infection, tumor development, lung inflammation, and fibrosis (Gordon and Martinez, 2010). Given the complex immunological milieu that characterizes each of these conditions, alternatively polarized macrophages are likely to encounter inflammatory stimuli as well (Fort et al., 2001; Ruffell et al., 2012). It has been shown that *in vitro* modeling of complex immunological microenvironment by IL-4 and IFN-γ co-stimulation leads to the attenuation of IFN-γ-induced transcriptional activation due to the effects of IL-4 on restrictive set of auxiliary transcription factors in mouse macrophages (Piccolo et al., 2017). These results suggest that alternatively polarized macrophages exhibit an altered responsiveness to inflammatory signals. The underlying crosstalk at the epigenomic and transcriptional levels remained largely unexplored. One of the effector functions of macrophages is the integration of different danger signals with NLRP3 inflammasome activation (Rathinam and Fitzgerald, 2016). Inflammasomes play key roles in the generation of secreted forms of proinflammatory IL-1β and IL-18 from their precursors. In parallel, macrophages undergo active NLRP3 inflammasome-dependent cell death termed "pyroptosis" (Rathinam and Fitzgerald, 2016). The integration of this process to inflammatory epigenomic signaling is also not known.

We sought to address these questions regarding the integration and regulation of the alternatively polarized macrophage phenotype by carrying out systemic genome-wide studies.

RESULTS

IL-4 Induces Transcriptional Activation and Repression via STAT6

We determined the STAT6-dependent IL-4-regulated genes in a time course in wild-type (WT) and *Stat6*$^{-/-}$ bone marrow-derived macrophages (BMDMs) using RNA-seq (Figure S1A). First, we examined the gene expression pattern of the 1,614 IL-4-regulated genes (Fc $\geq$ 2, p value $<$ 0.05) and identified four IL-4-induced gene expression clusters based on expression dynamics and fold induction (Figures 1A and S1B; Table S1). We also found that a high proportion of IL-4-responsive genes (39%) were repressed. Repression by IL-4 was observed after 3 hr and remained attenuated at later time points (6, 24 hr) (Figure 1A, cluster E; Table S1). IL-4-mediated repression is dependent on STAT6 (Figure 1B). For validation, we measured the mRNA level of six IL-4-repressed (*Abca1*, *Clec4d*, *Fos*, *Tlr2*, *Cd14*, and *Nlrp3*) and three activated (*Klf4*, *Hbegf*, and *Edn1*) genes with RT-qPCR, and we confirmed the IL-4-mediated and STAT6-dependent regulation (Figures 1C and S1C).

Filarial nematode infection is associated with the accumulation of alternatively polarized macrophages, exhibiting elevated expression of *Ym1* and *Fizz1*/RELM-α (Anthony et al., 2006). In order to determine whether transcriptional repression in response to alternative polarization signals occurs *in vivo*, we compared the gene expression profile of peritoneal macrophages from *Brugia malayi* nematode-implanted mice (Ne-Mac) and thioglycollate-elicited peritoneal macrophages (Thio-Mac) utilizing publicly available RNA-seq data (Thomas et al., 2012). Gene set enrichment analysis (GSEA) showed that the *in vitro* IL-4-repressed gene set was significantly enriched (FDR q-value $<$ 0.1, NER: -2.38) among the genes that were downregulated in response to nematode infection in peritoneal macrophages (Figure 1D). In addition, all selected IL-4-STAT6-repressed genes were significantly downregulated during *Brugia malayi*-induced *in vivo* alternative macrophage polarization compared to thioglycollate-elicited peritoneal macrophages (Figure 1E).

Next, we determined whether IL-4-STAT6 signaling represses gene expression at the transcriptional or post-transcriptional level. We assessed the immediate early effect of IL-4 on two serine phosphorylated forms of RNA polymerase II (RNAPII), the active histone mark H3K27Ac using chromatin immunoprecipitation sequencing (ChIP-seq), and nascent RNA expression by Global Run-On sequencing (GRO-seq) after 1 hr of exposure. Elongation-specific RNAPII-pS2 ChIP-seq revealed 5,931 gene bodies, exhibiting significantly changing read enrichments (3,008 downregulated and 2,923 upregulated, p $\leq$ 0.1) (Figure S2A and Table S2). RNAPII-pS2 binding showed positive correlation with transcription initiation-specific RNAPII-pS5 binding, H3K27Ac enrichment, and nascent RNA expression at the gene bodies (Figure S2B). Importantly, the gene bodies of IL-4-repressed genes (cluster E) showed attenuated RNAPII-pS2, RNAPII-pS5, and H3K27Ac enrichment and nascent RNA expression (Figures 2A, 2B, and S2C). In contrast, IL-4-dependent induction of RNAPII-pS2, RNAPII-pS5, and H3K27Ac enrichment as well as nascent RNA expression was detected at gene bodies of IL-4-induced genes (clusters A–C) (Figures 2A, S2C, and S2D). These results indicate that IL-4-STAT6 signaling directly represses gene expression, primarily at the transcriptional level during alternative macrophage polarization *in vitro* and *in vivo*.

IL-4-Activated STAT6 Binding Is Required for Transcriptional Repression

We also determined the STAT6 cistrome using a time course of 1, 6, and 24 hr of IL-4 stimulation (Figure S1A). STAT6 binding was negligible in unstimulated BMDMs (Figure 3A), but as little as 1 hr of stimulation dramatically induced the binding of STAT6, which was followed by a decline after 24 hr (Figure 3A). Comparing the STAT6 cistrome (20,119 genomic regions in IL-4-stimulated cells) to the RNAPII-pS5-positive genomic regions revealed that 60.5% of STAT6 peaks overlapped with the union of those genomic regions bound by RNAPII-pS5 in resting or IL-4-treated BMDMs (Figure 3B), suggesting that transcription could be directly regulated by STAT6 at these sites. Therefore, we next classified the RNAPII-pS5-positive STAT6 peaks based on IL-4-dependent regulation of RNAPII-pS5 binding, and we

(D) GSEA analysis of IL-4-repressed genes (*in vitro*) against a ranked list of genes regulated in the *Brugia malayi*-implanted mice-derived macrophages (Ne-Mac) compared to the intraperitoneal thioglycollate-administrated mice-derived peritoneal macrophages (Thio-Mac).

(E) Expression of IL-4-repressed genes in the *Brugia malayi*-implanted mice-derived macrophages (Ne-Mac) and the intraperitoneal thioglycollate-administrated mice-derived peritoneal macrophages (Thio-Mac). Reads per kilobase per million values (RPKM) are presented as the mean and SD of three individual animals per group quantified by RNA-seq. *p $<$ 0.05, **p $<$ 0.01, ***p $<$ 0.001.

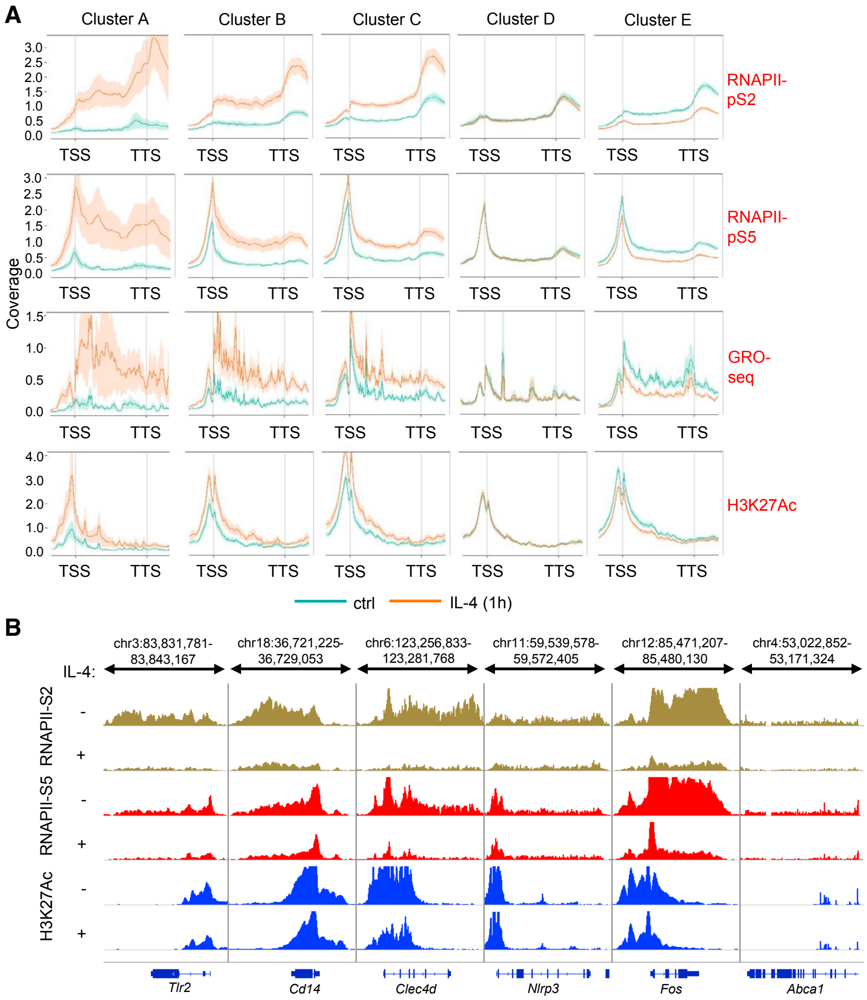

Figure 2. Changes in RNAPII and H3K27Ac Enrichments as well as Nascent RNA Transcription Are Immediate Early Markers of IL-4-STAT6-Regulated Transcription

(A) Metagene plot of RNAPII-pS5-, RNAPII-pS2-, and H3K27Ac-specific ChIP-seq enrichments and GRO-seq signals on the gene bodies of regulated gene clusters (Figure 1A) in the presence of IL-4 in WT BMDMs (TSS, transcription start site; TTS, transcription termination site). Coverage is defined as read count per million mapped reads. Data (H3K27Ac, RNAPII-pS2, and RNAPII-pS5) are combined from two independent biological replicates.

(B) H3K27Ac, RNAPII-pS5, and RNAPII-pS2 ChIP-seq signals at the selected IL-4-repressed gene bodies. ChIP-seq signals are visualized by the Integrative Genomics Viewer. Data are representative of two independent biological replicates. BMDMs were treated with IL-4 for 1 hr.

divided the STAT6-bound genomic regions into three different clusters: "repressor," "neutral," and "activator" STAT6 peak clusters (Figure 3C and Table S3). We noted that repressor and neutral STAT6 peaks showed typically lower occupancies if compared to the IL-4-induced RNAPII-pS5-associated activator STAT6 peaks (Figure S3A). Interestingly, IL-4-dependent regulation of RNAPII-pS2 binding as well as H3K27Ac enrichments showed similar patterns to RNAPII-pS5 in all three STAT6 clusters (Figures 3C and 3D). These findings support the conclusion that IL-4-activated STAT6 can be associated with either transcriptional activation or repression at different genomic loci.

Next we assigned STAT6-bound genomic regions to genes in order to assess the correlation between IL-4-repressed enhancer activity (RNAPII-pS5 by ChIP-seq) and gene expression (mRNA by RNA-seq). For this analysis, we predicted the sub-topologically associated domains (subTADs) in which gene regulation by STAT6 might take place, using CTCF and RAD21 ChIP-seq datasets from BMDM, utilizing a previously described algorithm (Daniel et al., 2014; Rao et al., 2014). As shown in Figure 3E, we found that repressor STAT6 peaks were tightly associated with the IL-4-repressed gene cluster (cluster E). In contrast, activator STAT6 peaks were associated with the immediate early IL-4-induced genes represented by clusters A–C (Figure 3E). These results suggest a tight connection between STAT6-dependent regulation of enhancer activity and neighboring gene expression in the same genomic compartment or transcription unit.

To understand the IL-4-STAT6 signaling-mediated transcriptional regulation in more detail, we carried out analyses on individual genes and enhancers. For the selected repressed and activated genes, we identified at least one STAT6-bound enhancer showing reduced and induced H3K27 acetylation and RNAPII binding, respectively (Figure 3F). Enhancer RNA (eRNA) expression is a reliable marker of enhancer activity (Natoli and Andrau, 2012). Therefore, we measured eRNA expression at the repressor and activator STAT6 peaks by RT-qPCR. The expression of eRNAs was regulated in a similar manner as the enrichment of RNAPII-pS5 and RNAPII-pS2 and changes of H3K27Ac levels at the repressor and activator STAT6 sites in WT BMDMs (Figures 3F, 3G, and S3B). Importantly, IL-4-mediated regulation of eRNA expression was abolished in the absence of STAT6 at the examined enhancers (Figures 3G and S3B). Taken together, these results show that IL-4-activated STAT6 is required for the transcriptional repression characterized by decreasing RNAPII binding, histone acetylation, and consequently enhancer activity.

STAT6 Binds to Repressed Sites in the Absence of a Canonical Binding Motif

In order to investigate whether the functional characteristics of STAT6 peaks (activator versus repressor) are influenced by their genomic localization and/or the DNA sequences they are associated with, we analyzed the genomic distribution of STAT6 peak clusters. We found only minor differences between the distinct STAT6 peak clusters regarding genomic localization

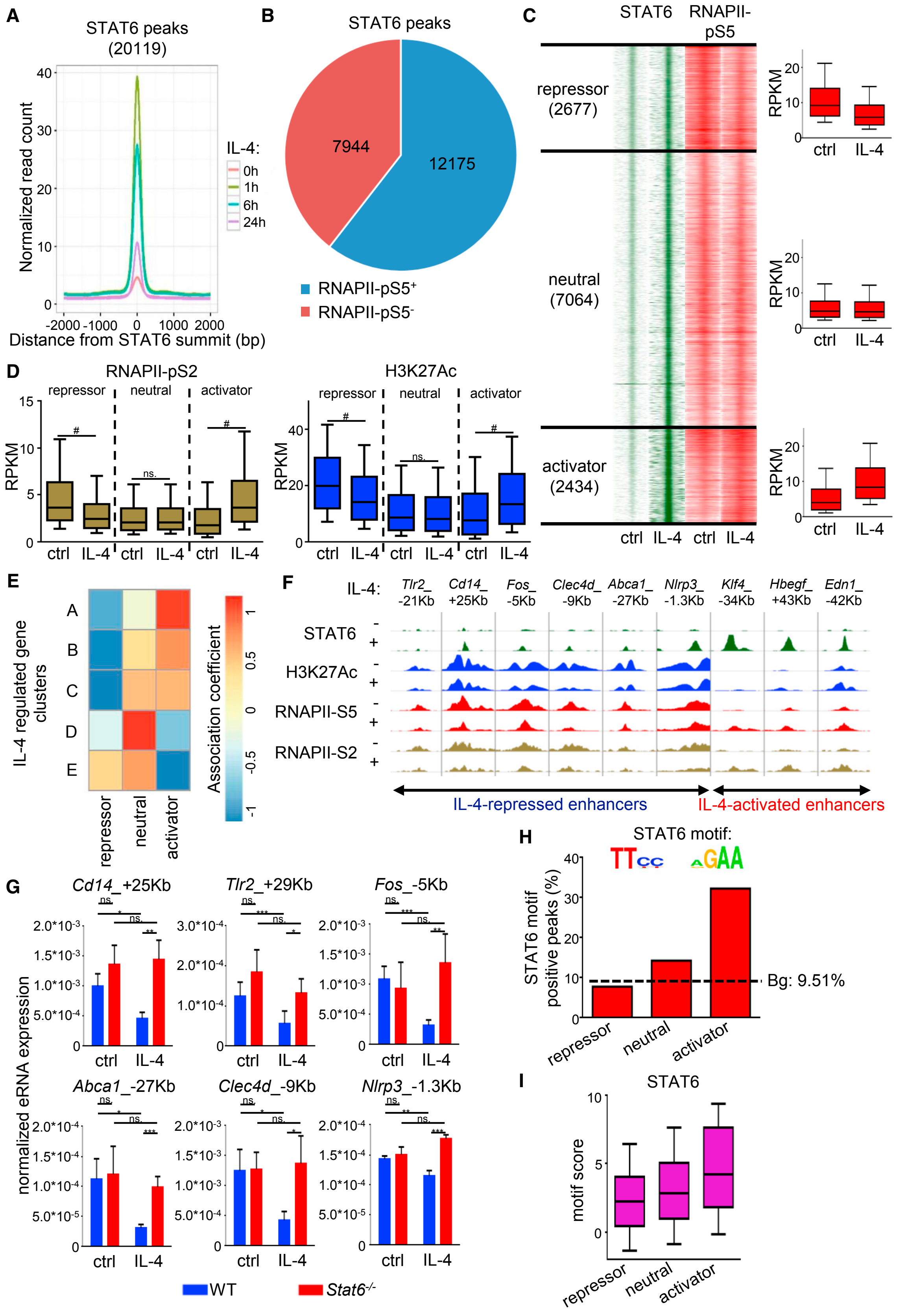

(legend on next page)

relative to genes (Table S4). The majority of STAT6 peaks were localized in intergenic and intronic regions in the genome in all three clusters, and only about 10% of STAT6 binding sites were detected in promoter-proximal regions (Table S4). We also examined the enrichment of active histone mark H3K4m1 at the STAT6-bound genomic regions using a publicly available ChIP-seq dataset (Ostuni et al., 2013). Although H3K4m1 enrichment was observed at more than 98% of STAT6-bound genomic regions, it was not influenced by IL-4 treatment (Table S4, Figure S3C). These findings suggest that STAT6 primarily binds enhancers and that the functional characteristics of different STAT6 peak clusters cannot be explained by their genomic localization relative to genes.

Next, we carried out *de novo* motif enrichment analysis of the sequences under the STAT6 peaks. PU.1, TRE, RUNX, and C/EBP motifs were enriched under all three clusters (Figure S3D). However, the canonical STAT6 motif was significantly underrepresented under repressor and neutral STAT6 peaks if compared to the activator STAT6 peaks (Figures 3H and S3D). Plotting the motif scores for PU.1, TRE, RUNX, and C/EBP revealed no significant differences between the different STAT6 peak clusters (Figure S3E). In contrast, motif score for STAT6 was lower in the repressor and neutral STAT6 peak clusters compared to the activator STAT6 peak cluster (Figure 3I). Considering that the presence of STAT6 is needed for repression (Figure 1B), these findings raise the possibilities that STAT6 is bound without direct DNA contact or that it recognizes non-canonical STAT6-binding motifs at repressed enhancers.

STAT6-Mediated Repression of Enhancer Activity Is Accompanied by Decreased Chromatin Accessibility and Lineage-Determining Transcription Factor Binding

We investigated whether chromatin accessibility is affected at the STAT6-bound genomic regions by performing Assay for Transposase-Accessible Chromatin using sequencing (ATAC-seq) experiments. Our genome-wide analyses showed increased chromatin accessibility at the activator STAT6-bound sites (Figure 4A), while significant reduction was detected in chromatin accessibility at the repressor STAT6-bound genomic regions (Figure 4A). These results suggest that both STAT6-mediated enhancer activation and repression are associated with the modification of chromatin structure during alternative macrophage polarization.

Chromatin openness determines enhancer activity in different cell types (Shlyueva et al., 2014). Moreover, binding of macrophage LDTFs, PU.1, JUNB, IRF8, and C/EBPα are associated with active enhancers in macrophages (Glass and Natoli, 2016). In addition, their binding motifs were among the most enriched transcription factor motifs under STAT6 peaks (Figure S3D). Therefore, we decided to determine whether IL-4-STAT6 signaling-mediated repression is associated with modified binding of LDTFs and examined their binding at repressed enhancers in the presence or absence of IL-4 using ChIP-seq. A high portion of the STAT6 cistrome overlapped with the examined LDTF cistromes except for JUNB, which showed moderated overlap (Table S4). Intriguingly, PU.1, JUNB, and C/EBPα binding was significantly decreased, while IRF8 binding was not modulated at the repressed STAT6-bound genomic regions after 1 hr IL-4 treatment in BMDMs (Figures 4B and S4A). In contrast, all four LDTFs showed significantly elevated binding at the IL-4-activated enhancers following IL-4 stimulation (Figures 4B and S4A). These findings suggest that IL-4-STAT6 signaling pathway modulates the binding of LDTFs at STAT6-activated and -repressed enhancers to opposite directions.

IL-4-STAT6 Signaling Pathway-Mediated Repression of Enhancers Is Characterized by an Altered p300:HDAC Ratio

The acetylation status and thus the activity of enhancers are tightly controlled by histone acetyltransferase (HAT) and histone deacetylase (HDAC) enzymes (Calo and Wysocka, 2013). Therefore, we examined the binding of the histone acetyltransferase p300 as well as classical histone deacetylases, including HDAC1, 2, and 3 at the STAT6-bound genomic regions after 1 hr of IL-4 exposure by ChIP-seq. We found that the majority of STAT6-bound genomic regions were either pre-loaded by p300 and classical HDACs or recruited these factors upon IL-4

Figure 3. RNAPII-pS5-Based Characterization of IL-4-Activated STAT6 Cistrome in Mouse Macrophages

(A) Histograms of the average coverage of STAT6 peaks at the indicated period of time following IL-4 treatment in WT BMDMs.

(B) Pie chart of the RNAPII-pS5-positive and -negative STAT6-bound regulatory regions, 1 hr of IL-4 stimulation.

(C) Read distribution plot of ChIP-seq intensities for STAT6 and RNAPII-pS5 around the summit of the detected STAT6 peaks in a 4 kb window (left). Clustering of STAT6-RNAPII-pS5 co-bound genomic regions was based on the usage of DiffBind analysis ($p \leq 0.05$). Boxplots of the average RPKM values for RNAPII-pS5 in each cluster (right). Boxes encompass the 25th to 75th percentile RPKMs. Whiskers extend to the 10th and 90th percentiles.

(D) Boxplots of RNAPII-pS2 and H3K27Ac read enrichments (RPKM) around the identified STAT6 peak clusters in WT BMDMs. Boxes encompass the 25th to 75th percentile RPKMs. Whiskers extend to the 10th and 90th percentiles.

(E) Heatmap of correlations between STAT6 peak (C) and IL-4-regulated gene clusters (Figure 1A) based on genomic proximity and functional chromatin domain prediction.

(F) Integrative Genomics Viewer snapshots of STAT6, H3K27Ac, RNAPII-pS5, and RNAPII-pS2 ChIP-seq signals on a set of IL-4-repressed and activated genomic loci.

(G) RT-qPCR measurements of eRNA expression at IL-4-repressed enhancers in WT and *Stat6*$^{-/-}$ macrophages.

(H) Bar plots showing the percentage of the STAT6 motif-positive STAT6 peaks in the clusters on (C). The STAT6 binding motif was used for targeted search. Dashed line indicates background (Bg: 9.51%).

(I) Boxplot of STAT6 motif scores at the functionally distinct STAT6 peak clusters. Boxes encompass the 25th to 75th percentile motif scores. Whiskers extend to the 10th and 90th percentiles.

BMDMs (B–D, F, and G) were treated with IL-4 for 1 hr. Data in (A)–(D) are combined from two independent biological replicates. Changes in (D) were considered significant at $p < 0.00001$ using paired t test and an average fold change cut off value of ≥ 1.15 was used between control and IL-4-treated samples. # means significant difference, n.s. indicates not significant change. Data in (F) are representative of two independent biological replicates. Data in (G) are representative of five individual animals per genotype from two independent experiments. *$p < 0.05$, **$p < 0.01$, ***$p < 0.001$, ns, not significant change. Error bars represent mean ± SD.

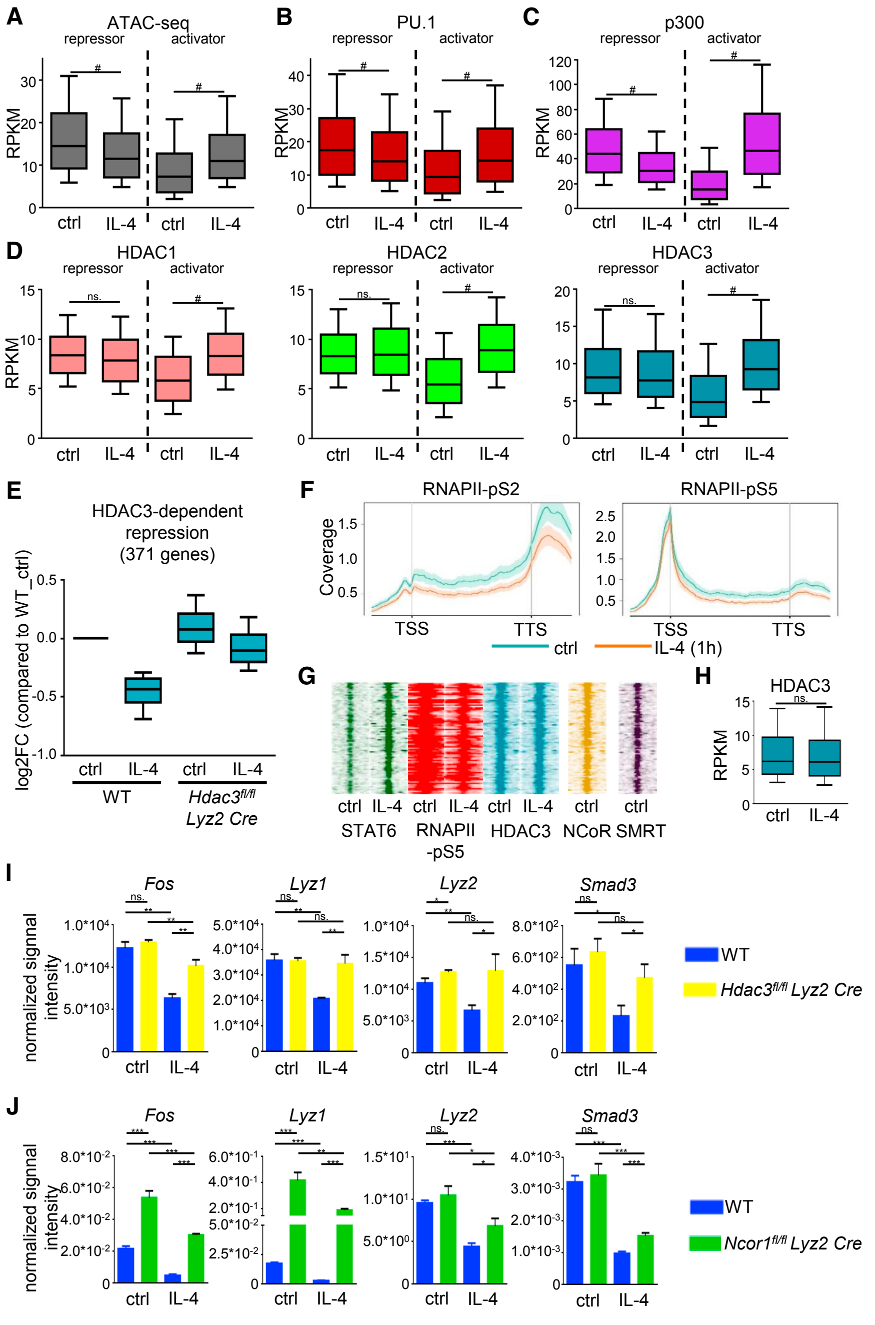

Figure 4. IL-4-Induced Changes at Repressor and Activator STAT6 Sites and the Role of HDAC3 in IL-4-STAT6-Mediated Repression

(A–D) Boxplots of ATAC-seq (A) and ChIP-seq (B–D) signals for PU.1 (B), p300 (C), HDAC1 (D), HDAC2 (D), and HDAC3 (D) on the repressor and activator STAT6 sites in WT BMDMs. Boxes encompass the 25th to 75th percentile RPKMs. Whiskers extend to the 10th and 90th percentiles.

(E) Boxplots of the expression of IL-4-HDAC3-dependent repressed genes in WT (n = 3) and *Hdac3*$^{fl/fl}$ *Lyz2 Cre* (n = 3) BMDMs using publicly available microarray results. Boxes encompass the 25th to 75th percentile changes. Whiskers extend to the 10th and 90th percentiles.

(F) Metagene plots of RNAPII-pS5 and RNAPII-pS2 signals at the gene bodies of IL-4-HDAC3-dependent repressed genes. Coverage is defined as read count per million mapped reads.

(G) Read distribution plot of ChIP-seq intensities for RNAPII-pS5, STAT6, HDAC3, NCoR, and SMRT around the summit of the detected STAT6 peaks at the IL-4-repressed enhancers (n = 325) in the subTADs of HDAC3-dependent repressed genes, 1 hr of IL-4 stimulation.

(H) Boxplot of the average HDAC3 binding intensity on the genomic regions (G). Boxes encompass the 25th to 75th percentile RPKMs. Whiskers extend to the 10th and 90th percentiles.

(I) Normalized microarray signal intensity of *Fos*, *Lyz1*, *Lyz2*, and *Smad3* in control or IL-4-stimulated WT and *Hdac3*$^{fl/fl}$ *Lyz2 Cre* BMDMs.

(J) RT-qPCR measurements of *Fos*, *Lyz1*, *Lyz2*, and *Smad3* expression in control or IL-4-stimulated WT and *Ncor1*$^{fl/fl}$ *Lyz2 Cre* iBMDMs.

BMDMs were treated with IL-4 for 1 hr (A–D, F–H) or 24 hr (E, I, and J). Data (A–D, F, and H) are combined from two independent biological replicates. Changes (A–D and H) were considered significant at $p < 0.00001$ using paired t test and an average fold change cut off value of ≥ 1.15 was used between control and IL-4-treated samples. # means significant difference, ns indicates not significant change. Data (I) represent the mean and SD of three independent biological replicates. *$p < 0.05$, **$p < 0.01$, ***$p < 0.001$, ns, no significant difference. Error bars represent means ± SD. Data (J) represent the mean and SD of three independent biological replicates. *$p < 0.05$, **$p < 0.01$, ***$p < 0.001$, ns, no significant difference. Error bars represent means ± SD.

stimulation (Table S4). The binding of p300 was significantly increased at STAT6-activated enhancers, but significantly reduced at STAT6-repressed enhancers upon IL-4 treatment (Figure 4C). Interestingly, genome-wide analysis of IL-4-modulated HDAC binding showed significantly enhanced HDAC1, 2, and 3 occupancy at STAT6-activated enhancers, while STAT6-repressed enhancers showed no effect to IL-4, but exhibited HDAC binding at the basal state (Figure 4D). Collectively, these results show that STAT6-repressed enhancers are bound by both HATs and HDACs at the steady state and that p300 binding is selectively reduced by IL-4, resulting in a changed equilibrium favoring HDAC activity.

The Presence of HDAC3 Is Required for IL-4-STAT6-Mediated Repression on a Subset of Genes

Direct interactions between classical HDACs and STAT transcription factors have been observed previously in numerous cell types influencing STAT-mediated direct transcriptional regulation (Icardi et al., 2012; Nusinzon and Horvath, 2003). In addition, HDAC3 has been shown to participate in the regulation of alternative macrophage polarization *in vitro* and *in vivo* (Mullican et al., 2011). Thus, we hypothesized that HDAC3, which is present at repressed enhancers (Figure 4D), might also contribute to IL-4-STAT6-induced repression. Therefore, we decided to examine the role of HDAC3 using a dataset from Mullican et al.

(2011). Applying K-mean clustering method, we found 1,628 IL-4-repressed genes ($p \leq 0.05$) in WT BMDMs (Figure S4B) and identified an IL-4-repressed gene cluster (cluster III, 371 genes) that showed attenuated repression in *Hdac3*$^{fl/fl}$ *Lyz2 Cre* BMDMs following IL-4 treatment (Figures 4E and S4B). Although the basal expression of these genes did not show major differences between WT and *Hdac3*$^{fl/fl}$ *Lyz2 Cre* BMDMs, the IL-4-induced repression was partially or completely abolished in the absence of HDAC3 (Figures 4E and S4B). In addition, enrichments of RNAPII-pS5 and RNAPII-S2 were reduced at these gene bodies after 1 hr of IL-4 treatment in WT BMDMs (Figure 4F). Interestingly, 325 STAT6-repressed enhancers were found within the subTADs of IL-4-HDAC3-repressed genes (Figure 4G). These enhancers were bound by HDAC3, but HDAC3 occupancy was not altered by IL-4 stimulation (Figures 4G and 4H). Our results indicate that HDAC3 is required for the IL-4-induced repression of a specific subset of genes.

Due to the fact that HDAC3 is one of the key components of NCoR and SMRT corepressor complexes (Karagianni and Wong, 2007), we decided to determine whether the NCoR-SMRT complex itself participates in IL-4-STAT6-HDAC3-mediated repression as well. First, we determined the occupancy of NCoR and SMRT at HDAC3-bound enhancers using ChIP-seq data generated by others (Barish et al., 2012). We found that the IL-4-STAT6-HDAC3-repressed enhancer set was bound by both NCoR and SMRT in unstimulated BMDMs (Figure 4G). Next, we investigated the requirement of NCoR in the IL-4-STAT6-HDAC3-mediated repression using *Ncor1*$^{fl/fl}$ *Lyz2 Cre* immortalized bone marrow-derived macrophages (iBMDMs). We selected four genes for this analysis (*Fos*, *Lyz1*, *Lyz2*, and *Smad3*) based on their IL-4-STAT6-HDAC3-dependent repression (Figures 4I, S4C, and S4D) and due to the fact that their enhancers were bound by HDAC3, NCoR, and SMRT (Figure S4C). Gene expression analysis showed that *Fos* and *Lyz1* were expressed at a significantly higher level in unstimulated iBMDMs in the absence of NCoR compared to WT iBMDMs, while the basal expression of *Lyz2* and *Smad3* were not affected by NCoR (Figure 4J). In addition, IL-4-mediated repression of these genes was diminished in *Ncor1*$^{fl/fl}$ *Lyz2 Cre* iBMDMs (Figures 4J and S4E). In contrast, the basal expression and IL-4-induced repression of HDAC3-independent genes were not affected by NCoR, except for *Abca1* (Figure S4F). Taken together, our findings suggest that IL-4-activated STAT6 mediates transcriptional repression via the NCoR-HDAC3 complex at a subset of genes representing one of the molecular mechanisms for STAT6-dependent transcriptional repression.

IL-4-STAT6-Mediated Direct Transcriptional Repression Affects the LPS-Induced Inflammatory Program of Macrophages

Next we were wondering whether the repressive action of IL-4-STAT6 leaves its footprint on the epigenome and affects the subsequent response of the cells to other stimuli. Using KEGG pathway analysis, we identified 12 signaling pathways whose overrepresentation was specific to IL-4-repressed genes (Figure S5A). NOD-like receptor signaling and Toll-like receptor signaling among the top hits, which are known to be two major regulators of the inflammatory response (Figure S5A; Chen et al., 2009; Takeda et al., 2003). In addition, upstream transcriptional regulator analysis with Ingenuity Pathway Analysis (IPA) software revealed that the LPS-activated p65 (RelA) is one of the most significantly inhibited transcriptional regulators upon IL-4 stimulation (Figure S5B). Interestingly, the majority of IL-4-STAT6-repressed genes included several members of NOD-like and Toll-like receptor signaling pathways showing attenuated mRNA expression following 24 hr of IL-4 stimulation and reduced STAT6 binding at the repressed enhancers (Figures 1A, 1B, S5C, and S5D). These results raised the possibility that IL-4 is able to modulate the subsequent inflammatory response of the macrophage epigenome via directly repressed enhancers following the dissociation of STAT6.

In order to determine whether prior activation of IL-4-STAT6 signaling is able to influence the inflammatory program of macrophages, we performed RNA-seq as well as RNAPII-pS5-, RNAPII-pS2-, and p65-specific ChIP-seq experiments on IL-4-pretreated and LPS-activated BMDMs (Figure 5A). Our global transcriptome analysis identified 1,350 LPS-activated genes ($p \leq 0.05$) in BMDMs (Figure 5B, Table S5). Intriguingly, 520 genes showed significantly attenuated ($p \leq 0.05$) LPS responsiveness following IL-4 pretreatment including the above examined *Tlr2*, *Cd14*, *Clec4d*, and *Nlrp3* (Figure 5B, Table S5). In addition, LPS-mediated induction of 686 genes was not influenced significantly by IL-4 pretreatment, while 144 genes showed significantly elevated LPS activation in IL-4-pretreated BMDMs (Figure 5B and Table S5), suggesting that the interaction between the two pathways is not a general interference or attenuation, but it takes place on selective genomic regions. Interestingly, 6 out of 12 IL-4-repressed signaling pathways were also significantly overrepresented among the genes that were less induced by LPS following IL-4 pretreatment, including NOD-like receptor and Toll-like receptor signaling pathways (Figure S5E). Next, we investigated whether IL-4-STAT6 signaling modulates LPS-dependent activation of inflammatory genes at the transcriptional or post-transcriptional level. We examined the IL-4- and LPS-regulated binding of RNAPII-pS2 at the gene bodies of LPS-induced genes using ChIP-seq. RNAPII-pS2 binding showed a similar pattern to "steady-state" mRNA level in all three gene expression clusters, suggesting that IL-4 pretreatment modulates LPS-induced gene expression primarily at the transcriptional level (Figure 5C). In order to investigate whether the IL-4-attenuated inflammatory response is STAT6 dependent, we measured the expression of *Tlr2*, *Cd14*, *Clec4d*, and *Nlrp3* in WT and *Stat6*$^{-/-}$ BMDMs after 24 hr of IL-4 pretreatment followed by 3 hr of LPS activation. As expected, IL-4 pretreatment failed to inhibit the LPS response of these genes in the absence of STAT6 (Figure 5D), raising the possibility that IL-4-activated STAT6 can directly modulate LPS responsiveness in macrophages via transcriptional repression of certain components of the inflammatory program.

To determine whether the crosstalk between IL-4-STAT6 signaling and inflammation-activated signaling pathways can also be observed at the enhancer level, we compared the IL-4-activated STAT6- and LPS-activated p65 cistromes in the subTADs of IL-4-attenuated LPS-responsive genes. 961 genomic regions were identified with overlapping STAT6 and p65 peaks revealing a partial overlap between the STAT6 and p65 cistromes (Figures 6A and 6B). Next, we decided to investigate whether IL-4-STAT6 and inflammatory signaling pathways are

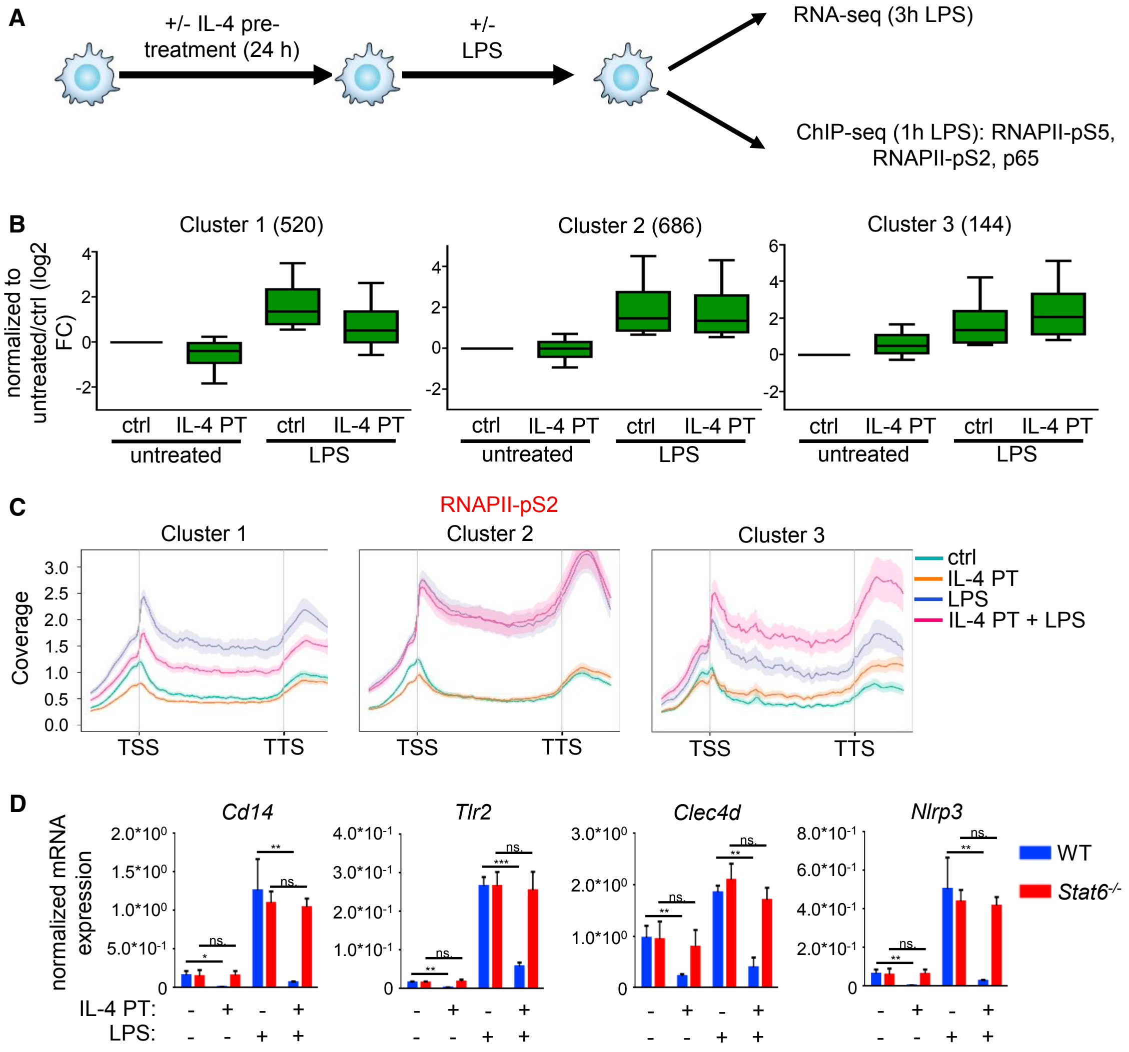

Figure 5. Selective Repression of LPS-Activated Inflammatory Program by IL-4-Activated STAT6

(A) Schematic representation of the experimental system.

(B) Boxplot of the fold changes of LPS-activated genes (RNA-seq). Clustering was based on the different LPS-induced gene expression effects on IL-4-pretreated and untreated BMDMs ($p < 0.05$). Data represent the average fold changes of three individual animals from two independent experiments. Boxes encompass the 25th to 75th percentile changes. Whiskers extend to the 10th and 90th percentiles.

(C) Metagene plot of RNAPII-pS2 signals over the gene bodies of the genes in the clusters (B). Coverage is defined as read count per million mapped reads. Data are combined from two independent biological replicates.

(D) RT-qPCR measurements of basal and LPS-induced expression of the inflammation-associated genes in IL-4-pretreated and unstimulated WT and *Stat6*$^{-/-}$ BMDMs. Data are cumulative of four individual animals per genotype from two independent experiments. *$p < 0.05$, **$p < 0.01$, ***$p < 0.001$, ns, no significant difference. Error bars represent means ± SD.

BMDMs were pretreated with IL-4 for 24 hr followed by LPS exposure for 3 hr (B and D) or 1 hr (C).

able to interact with each other using RNAPII-specific ChIP-seq analysis. 641 out of 961 genomic regions were associated with significantly elevated RNAPII-binding following LPS activation (Figure 6B). Intriguingly, 70% (448/641) of LPS-activated enhancers showed significantly reduced basal and LPS-induced RNAPII binding after 24 hr of IL-4 pretreatment (Figures 6B–6E). To further explore the mechanism of IL-4-STAT6-attenuated inflammatory responsiveness, we determined LPS-induced p65 binding at this enhancer set in IL-4-pretreated and unstimulated BMDMs. Based on p65 binding, we could identify two subsets of these enhancers including IL-4-insensitive and IL-4-attenuated p65 binding-associated enhancers (Figures 6E and 6F). LPS-induced p65 binding was significantly reduced at 74 IL-4-repressed enhancers, while IL-4-repressed inflammatory response was not associated with modulated p65 binding at 374 enhancers (Figures 6E and 6F). To investigate the STAT6 dependency of IL-4-repressed enhancer activity and p65 binding, we selected three enhancers for both analyses. RT-qPCR-based eRNA expression analysis confirmed IL-4-mediated and STAT6-dependent repression of basal and

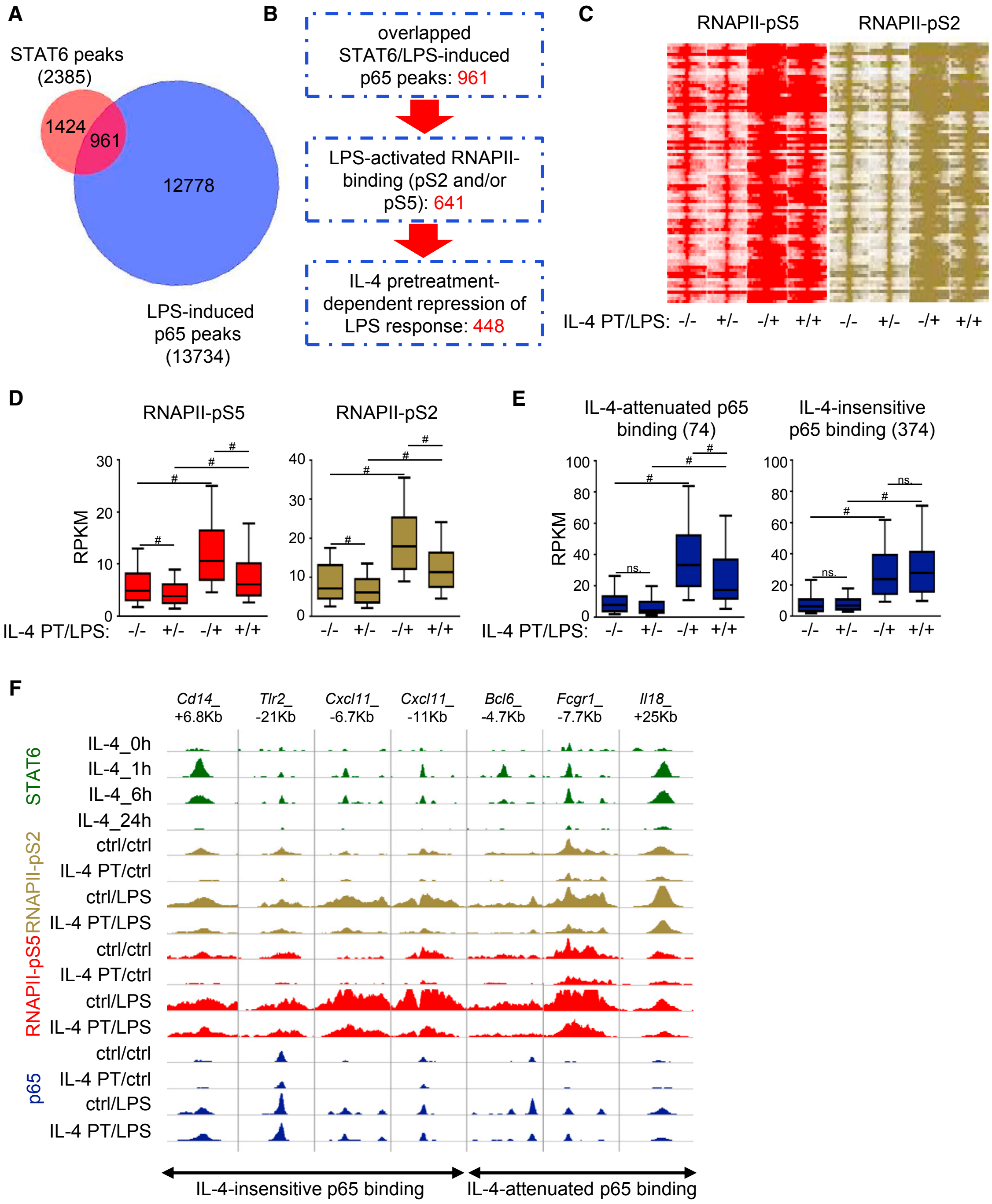

Figure 6. Attenuated Inflammatory Response Is Conferred by the Repressive Action of IL-4-STAT6 Signaling on a Subset of Enhancers

(A) Venn diagram of the overlap between the STAT6-bound regulatory regions associated to IL-4-inhibited LPS-responsive genes and the LPS-activated p65 cistrome.

(B) Flowchart of the identification of IL-4-repressed, LPS-inducible inflammatory enhancers. Significant changes in RNAPII binding were identified by DiffBind analysis ($p \leq 0.05$).

(C) Read distribution plot of ChIP-seq intensities for RNAPII-pS5 and RNAPII-pS2 around the summit of STAT6 peaks on the identified 448 overlapping STAT6 and p65-bound regulatory elements exhibiting IL-4-dependent attenuation of LPS response.

(D) Boxplot of the average coverage (RPKM) for RNAPII-pS5 and RNAPII-pS2 binding at the regulatory regions presented on the read distribution plot on (C), exhibiting attenuated LPS response in IL-4-pretreated BMDMs. Boxes encompass the 25th to 75th percentile RPKMs. Whiskers extend to the 10th and 90th percentiles.

(E) Boxplot of the average coverage (RPKM) for p65 binding at the regulatory regions presented on the read distribution plot on (C), exhibiting attenuated LPS response in IL-4-pretreated BMDMs. Regulatory regions showing repressed (left) and not influenced (right) p65 binding are shown. Boxes encompass the 25th to 75th percentile RPKMs. Whiskers extend to the 10th and 90th percentiles.

(legend continued on next page)

LPS-induced activity at the selected enhancers (Figure S6A). By performing ChIP-qPCR on the selected IL-4-reduced p65 binding-associated enhancers, we confirmed that IL-4-dependent reduction of LPS-induced p65 binding was completely abolished in the absence of STAT6 (Figure S6B).

Taken together, these findings suggest that the activation of IL-4-STAT6 signaling is able to attenuate the inflammatory response of macrophages through selective, direct repression of a distinct LPS-activated enhancer set.

IL-4-Mediated Repression of Inflammatory Response Results in Attenuated Inflammasome Activation, Decreased IL-1β Production, and Pyroptosis

Genes showing antagonistic regulation by IL-4 and LPS were mostly associated with inflammation-associated pathways, including NOD-like and Toll-like receptor signaling (Figure S5E). It is known that NOD-like receptors are required for inflammasome activation leading to IL-1β secretion and inflammasome-associated cell death, pyroptosis (Rathinam and Fitzgerald, 2016). As shown above, IL-4-STAT6 was able to reduce the basal and LPS-induced expression of a key inflammasome component, *Nlrp3* (Figures 1C and 5D). Therefore, we investigated whether IL-4-STAT6 signaling is able to influence IL-1β production and pyroptosis. First, we examined the transcriptional regulation of *Il1b* expression in IL-4-pretreated and LPS-activated BMDMs. Reduced basal and LPS-induced RNAPII-pS2 and RNAPII-pS5 binding at *Il1b* gene body as well as *Il1b* mRNA expression were found in IL-4-pretreated BMDMs (Figures 7A and 7B). Moreover, *Il1b*_−9.7Kb enhancer located within the predicted subTAD of *Il1b* showed antagonistic regulation by LPS and IL-4 (Figure 7A). Intriguingly, LPS-induced p65-binding at the STAT6-bound *Il1b*_−9.7Kb enhancer (in case of one out of two p65 peaks) was partially attenuated by IL-4-STAT6 signaling pathway similarly to *Fcgr1*_−7.7Kb and *Il18*_+25Kb enhancers described above (Figures 7A and 7C). Accordingly, IL-4-dependent repression of basal and LPS-induced eRNA expression was observed at the *Il1b*_−9.7Kb enhancer (Figure 7D). The IL-4-dependent repression of basal and LPS-induced *Il1b* mRNA and eRNA expression were completely abolished in the absence of STAT6 (Figures 7B and 7D). LPS-induced NLRP3 and pro-IL-1β expression were also attenuated at the protein level by IL-4-STAT6 signaling (Figures 7E and S7A), while the expression of other inflammasome components including proCaspase-1 and ASC was not altered following IL-4 and LPS stimulation of BMDMs (Figures 7E and S7A).

In order to determine whether IL-4-dependent repression of *Nlrp3* expression is associated with decreased inflammasome activity, we analyzed the LPS and ATP-induced Caspase-1 activity in IL-4-pretreated and unstimulated mouse BMDMs using laser scanning cytometry. As expected, Caspase-1 activation in WT BMDMs was induced dramatically by LPS and ATP costimulation (Figure 7F). Interestingly, LPS- and ATP-induced Caspase-1 activity was reduced significantly following 24 hr of IL-4 pretreatment, which was completely dependent on STAT6 (Figure 7F). To assess the functional consequence of IL-4-STAT6-dependent reduction of inflammasome activation, we measured the secretion of IL-1β in the presence of LPS and ATP in IL-4-pretreated and untreated WT and *Stat6*$^{-/-}$ BMDMs. IL-1β secretion was induced dramatically in WT and *Stat6*$^{-/-}$ BMDMs following LPS treatment (Figure 7G). However, LPS-dependent induction of IL-1β secretion was partially inhibited by IL-4 pretreatment in a STAT6-dependent manner (Figure 7G). Next, we investigated the role of IL-4-STAT6 signaling pathway in inflammasome activation-induced macrophage cell death, pyroptosis. A hallmark of pyroptosis is the insertion of pores into the plasma membrane that can be detected via LDH activity measurement from BMDM supernatants and propidium iodide (PI) staining. IL-4 pretreatment was able to effectively inhibit both LPS-induced LDH release and PI uptake in WT, but not in *Stat6*$^{-/-}$ BMDMs (Figures 7H and 7I).

Finally, we determined whether the inflammatory responsiveness of *Nlrp3* and *Il1b* is influenced *in vivo* by nematode infection triggered alternative macrophage activation. Therefore, we infected mice with *Heligmosomoides polygyrus* (*H. polygyrus*) and injected LPS or *Salmonella* Typhimurium into the peritoneal cavity 9 days after nematode infection. As expected, the number of the M2 macrophage marker Ym1-positive macrophages was highly induced in peritoneal macrophages of *H. polygyrus*-infected mice, confirming alternative macrophage activation following nematode infection (Figure S7B; Rückerl et al., 2017). In addition, inflammatory marker NOS2-positive macrophage number was dramatically elevated in control and *H. polygyrus*-infected mice following LPS injection or *Salmonella* Typhimurium infection, showing the emergence of infection (Figure S7B). Although *Nlrp3* was not induced in the applied experimental system by LPS injection or *Salmonella* Typhimurium infection, steady-state expression was significantly inhibited by *H. polygyrus* infection, and the inhibitory effect of nematode infection was sustained in the presence of inflammatory stimuli (Figure 7J). Nematode infection did not result in *Il1b* expression by peritoneal macrophages but both LPS injection and *Salmonella* Typhimurium infection resulted in a robust induction of *Il1b* expression. This elevated expression was significantly diminished in macrophages from *H. polygyrus*-infected mice (Figure 7J).

These results suggest that prior *in vitro* or *in vivo* alternative macrophage polarization are able to restrain the subsequent inflammatory response of macrophages, including inflammasome activation and IL-1β secretion as well as pyroptosis due to direct repression of *Nlrp3* and *Il1b* gene expression by IL-4-STAT6 signaling.

DISCUSSION

We unraveled an unsuspected repressor activity of the macrophage polarizing transcription factor, STAT6. We identified the

(F) Genome browser views of the IL-4 repressed regulatory regions showing attenuated LPS response in IL-4-pretreated BMDMs. ChIP-seq signals for RNAPII-pS5, RNAPII-pS2, and p65 are shown.

BMDMs were pretreated with IL-4 for 24 hr followed by 1 hr LPS exposure (A–E). Data (A–E) are combined from two independent biological replicates. Changes (D, E) were considered significant at $p < 0.00001$ using paired t test and an average fold change cut off value of $\geq$ 1.15 was used between control and IL-4-treated samples. # means significant difference, ns indicates not significant difference. Data (F) are representative of two independent biological replicates.

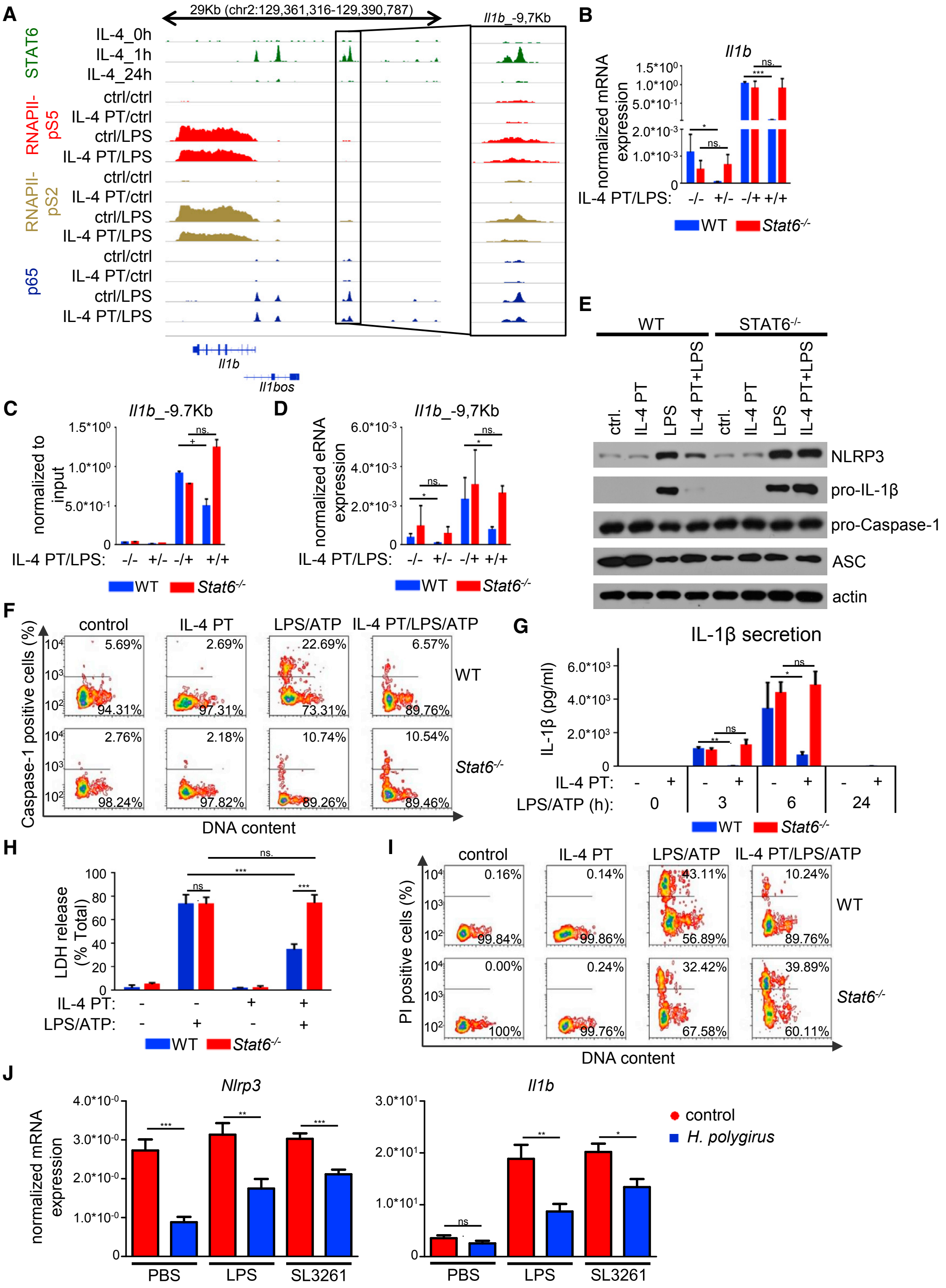

Figure 7. IL-4 Pretreatment Dampens the Inflammatory Response of Macrophages, Including Inflammasome Activation, IL-1β Production, and Pyroptosis

(A) Genome browser view of STAT6-, RNAPII-pS5-, RNAPII-pS2-, and p65-specific ChIP-seq signals on the *Il1b* locus. Data are representative of two independent biological replicates.

(legend continued on next page)

target enhancers of repressor STAT6, as well as the major components of the repressive mechanisms and its biological consequences.

Macrophages exhibit great functional diversity and the ability to undergo rapid reprogramming depending on the changing molecular milieu in physiological and pathological conditions (Mosser and Edwards, 2008; Rückerl et al., 2017). The epigenomic and molecular mechanisms of transcriptional activation by inflammatory mediators, cytokines, and lipids have been intensively studied in macrophages (for a review see Glass and Natoli, 2016). In contrast, the mechanistic background of macrophage polarization signal-mediated transcriptional repression is less understood, though LPS-reduced nascent RNA expression was described (Bhatt et al., 2012). In addition, LPS- and TNFα-dependent reduction of enhancer activity was also observed in macrophages and adipocytes in the absence of inflammation-activated NF-κB-p65 binding (Hah et al., 2015; Schmidt et al., 2015). In contrast, we show that the IL-4-STAT6 signaling pathway represses a significant portion of macrophage transcriptome via STAT6-bound enhancers, providing evidence that direct transcriptional repression also occurs during alternative macrophage polarization.

Although the global transcriptional repressor activity of the key TFs for macrophage polarization (NF-κB, AP-1, and STATs) is not fully understood, several other SDTFs have been shown to possess distinct repressor activities. Ligand-activated nuclear receptors including PPARs and LXRs are able to reduce the activity of inflammatory SDTFs through transrepression carried out by direct protein-protein interactions without direct DNA binding (Glass and Saijo, 2010). It has also been described that a specific NF-κB-binding motif is present in the promoter regions of tolerogenic inflammatory genes regulating LPS tolerance via recruitment of the p50-NCoR-HDAC3 repressosome (Yan et al., 2012). Our findings show that repressed enhancers exhibit lower STAT6 occupancy and underrepresented STAT6 *de novo* motifs compared to activated enhancers. These observations suggest that STAT6 acts as a transcriptional repressor either (1) without direct DNA binding or (2) by recognizing non-canonical STAT6-binding motifs. It remains to be identified which mechanism is at play and, if STAT6 binding to the DNA is indirect, which DNA-bound factor interacts with STAT6. Our motif analysis suggests that the lineage-determining factors (PU.1 and C/EBPα) are the most likely candidates though PU.1 and C/EBPα binding also decreased at STAT6-repressed enhancers. Thus this requires further investigations.

STAT6-mediated repression appears to be distinct from other repressive mechanisms. Histone acetylation and gene expression tightly depends on the cofactor composition (HAT:HDAC ratio) (Calo and Wysocka, 2013). Several findings show the extensive participation of co-repressor proteins and HDACs in the inhibition of transcriptional activation by cytokine-activated STAT proteins (Icardi et al., 2012). However, the molecular mechanisms of STAT6-mediated direct transcriptional repression following IL-4 activation have not been described. Our cistromic studies show diminished p300 binding at STAT6-repressed enhancers in IL-4-exposed macrophages, suggesting that reduced p300 binding is likely to be a key mechanism in the IL-4-STAT6 signaling pathway-mediated transcriptional repression. In addition, STAT6-repressed enhancers were occupied by HDAC1, 2, and 3, though their binding was not altered following IL-4 stimulation. Nevertheless, our findings show evidence for the participation of NCoR-HDAC3 corepressor complex in the IL-4-STAT6-induced transcriptional repression in a distinct subset of IL-4-repressed genes. Our data suggest that either the changed equilibrium between HATs and HDACs or perhaps the activity of HDAC3 are regulated upon IL-4 stimulus. Potential mechanisms for the latter include posttranslational modifications and allosteric regulation. The mechanisms of non-HDAC3-dependent repression also remain to be identified.

Alternatively, polarized macrophages are required for effective protection against different nematode infections reducing parasite number and inhibiting nematode-induced tissue damage (Allen and Sutherland, 2014). However, nematode

(B) RT-qPCR-based measurement of basal and LPS-induced *Il1b* expression in IL-4-pretreated and unstimulated WT and *Stat6*$^{-/-}$ BMDMs. Data are cumulative of four individual animals per genotype from two independent experiments. *p < 0.05, **p < 0.01, ***p < 0.001, ns, no significant change. Error bars represent means ± SD.

(C) ChIP-qPCR measurement of p65 binding at *Il1b*_−9.7Kb enhancer from WT and *Stat6*$^{-/-}$ BMDMs. Data represent the mean and SD of two biological replicates. $^{+}$p < 0.1, ns, not significant change.

(D) RT-qPCR measurement of basal and LPS-induced *Il1b*_−9.7Kb eRNA expression in IL-4-pretreated and unstimulated WT and *Stat6*$^{-/-}$ BMDMs. Data are cumulative of four individual animals per genotype from two independent experiments. *p < 0.05, **p < 0.01, ***p < 0.001, ns, not significant change. Error bars represent means ± SD.

(E) Western blot determination of basal and LPS-regulated Nlrp3, pro-IL-1β, pro-Caspase1, ASC, and β-actin expression in IL-4-pretreated and unstimulated WT and *Stat6*$^{-/-}$ BMDMs. Data are representative of five individual animals per genotype from two independent experiments.

(F) Contour map representation of laser-scanning imaging cytometry of Caspase-1 activity in WT and *Stat6*$^{-/-}$ BMDMs. Data are representative of two independent experiments.

(G) ELISA measurement of IL-1β secretion in IL-4-pretreated and unstimulated WT and *Stat6*$^{-/-}$ mouse BMDMs. Data represent the mean and SD of three individual animals. *p < 0.05, **p < 0.01, ***p < 0.001, ns, no significant change.

(H) Lactate dehydrogenase activity assay measurement of LPS and ATP co-stimulation-induced LDH release in IL-4-pretreated and unstimulated WT and *Stat6*$^{-/-}$ BMDM supernatants. LDH release expressed as the percentage of Triton X-100-liberated total LDH release. Data represent the mean and SD of three individual animals. *p < 0.05, **p < 0.01, ***p < 0.001, ns, no significant change.

(I) Contour map representation of laser-scanning imaging cytometry analysis of PI-labeled WT and *Stat6*$^{-/-}$ BMDMs. Data are representative of two independent experiments.

(J) Basal, LPS, and *Salmonella* Typhimurium (SL3261)-induced expression of Nlrp3 and *Il1b* expression in naive and *Heligmosomoides polygyrus* (*H. polygyrus*)-infected mice-derived peritoneal macrophages. Each data point represents the mean and SD of five to six individual animals per group. *p < 0.05, **p < 0.01, ***p < 0.001, ns, no significant change.

BMDMs were pretreated with IL-4 for 24 hr followed by LPS exposure for 1 hr (A and C), 3 hr (B, D, E, F, H, and I), or the indicated period (G).

infection-induced Th2 cell-type inflammation can also influence the immune response against other pathogens and the prevalence of autoimmune or inflammatory diseases (Elliott and Weinstock, 2012; Rückerl et al., 2017). Therefore, a better understanding of the potential interactions between Th2 and Th1 cell-type inflammation-activated signaling pathways has a great importance in macrophage biology and also in immune-inflammatory pathologies. It has been recently published that the IL-4-STAT6 signaling pathway can partially suppress IFN-γ-induced transcriptional program in macrophages following IL-4 and INF-γ co-treatment (Piccolo et al., 2017). However, our findings provide evidence that IL-4-STAT6 signaling pathway induces epigenetic changes that persist following the release of STAT6 from the DNA, leading to attenuated activation of inflammatory enhancers. The consequence of IL-4 priming-induced repression of inflammatory enhancers is the decreased responsiveness to inflammatory signals via diminished basal and LPS-induced expression of several components of Toll-like and Nod-like receptor signaling pathways. In addition, the majority of IL-4-STAT6-repressed genes show diminished expression in macrophages derived from *Brugia malayii*-implanted mice compared to thioglycollate-elicited macrophages. This formally suggests that alternative polarization likely induces partial desensitization of macrophages to further inflammatory signals *in vivo*. Accordingly, M2-type macrophages have been shown to protect mice against chemically induced colitis and a growing body of evidence indicates that clinically controlled helminth infection is able to ameliorate inflammatory bowel disease (IBD) (Weinstock and Elliott, 2013).

These studies suggest that complex bidirectional interactions exist between different polarization signals that determine the overall sensitivity and responsiveness of macrophages toward environmental stimuli. Our findings provide insights into this cross-talk at the level of individual enhancers and raise the intriguing possibility that IL-4-STAT6 signaling, through direct transcriptional repression of inflammatory enhancers, induces desensitization of macrophages to microbial-, stress-, and damage-associated endogenous signals.

STAR★METHODS

Detailed methods are provided in the online version of this paper and include the following:

- KEY RESOURCE TABLE
- CONTACT FOR REAGENT AND RESOURCE SHARING
- EXPERIMENTAL MODEL AND SUBJECT DETAILS
  - Mice
  - Bone Marrow-Derived Macrophages
  - Immortalization of mouse bone marrow-derived macrophages
- METHOD DETAILS
  - RNA-seq
  - ChIP-seq and ChIP-qPCR
  - ATAC-seq
  - GRO-seq
  - Real-Time Quantitative PCR for enhancer RNA and mRNA detection (qPCR)
  - LDH release
  - Measurement of IL-1β production
  - Western Blot analysis
  - Laser Scanning Cytometry
  - *In vivo* infection model
  - Flow cytometry
- QUANTIFICATION AND STATISTICAL ANALYSIS
  - RNA-seq analysis
  - ATAC-seq, ChIP-seq and GRO-seq analyses
  - Domain prediction
  - Statistical analysis
- DATA AND SOFTWARE AVAILABILITY

SUPPLEMENTAL INFORMATION

Supplemental Information includes seven figures and seven tables and can be found with this article online at https://doi.org/10.1016/j.immuni.2017.12.010.

ACKNOWLEDGMENTS

We thank Ms. Cseh, Szalka, Kriston, and Beladi for technical assistance and Dr. Szatmari and members of the Nagy laboratory for discussions and comments on the manuscript. This work was supported by the Hungarian Scientific Research Fund (K100196, K111941, and K116855 to L.N. and K109429 to S.B.) and by SBP Medical Discovery Institute to L.N. Library preparation and bioinformatic analysis were performed at the Genomic Medicine and Bioinformatic Core Facility of the University of Debrecen. RNA sequencing was performed at the Centre National de Genotypage (CNG) Evry, by S. McGinn, A. Boland, D. Lechner, and M.T. Bihoreau and supported by the European Sequencing and Genotyping Infrastructure (funded by the European Commission, FP7/2007-2013) under no. 26205 (ESGI) to L.N., and also at the Analytical Genomics Core Facility at the SBP. Z.C. was supported by funding from the European Union, the European Social Fund, and the State of Hungary (TÁMOP 4.2.4. A/2-11-1-2012-0001 "National Excellence Program"), B.D. by the American Heart Association (AHA) postdoctoral fellowship (17POST33660450), J.E.A. by the Medical Research Council UK (MR/K01207X/1), S.B. by a Bolyai and a Szodoray Postdoctoral Fellowship, and M.M.B. by the ÚNKP-17-4 New National Excellence Program of the Ministry of Human Capacities. The funders had no role in study design, data collection and analysis, decision to publish, or preparation of the manuscript.

AUTHOR CONTRIBUTIONS

L.N. and Z.C. directed the study and L.N., Z.C., and B.D. wrote the paper. Z.C., B.D., M.K., M.P., M.M.B., I.C.-M., Z.S., B.N., S.P., C.B., Z.B., I.G.S., S.B., D.R., and J.E.A. performed the experiments. A.H. designed the bioinformatic approaches and A.H., G.N., and L.S. analyzed and integrated the NGS data. S.S. and J.-F.D. directed sequencing. All authors provided input into data analysis and approved the final version of the manuscript.

DECLARATION OF INTERESTS

The authors declare no competing interests.

Received: September 7, 2017
Revised: November 9, 2017
Accepted: December 11, 2017
Published: January 16, 2018

REFERENCES

Allen, J.E., and Sutherland, T.E. (2014). Host protective roles of type 2 immunity: parasite killing and tissue repair, flip sides of the same coin. Semin. Immunol. *26*, 329–340.

Anthony, R.M., Urban, J.F., Jr., Alem, F., Hamed, H.A., Rozo, C.T., Boucher, J.L., Van Rooijen, N., and Gause, W.C. (2006). Memory T(H)2 cells induce

alternatively activated macrophages to mediate protection against nematode parasites. Nat. Med. *12*, 955–960.

Barish, G.D., Yu, R.T., Karunasiri, M.S., Becerra, D., Kim, J., Tseng, T.W., Tai, L.J., Leblanc, M., Diehl, C., Cerchietti, L., et al. (2012). The Bcl6-SMRT/NCoR cistrome represses inflammation to attenuate atherosclerosis. Cell Metab. *15*, 554–562.

Barta, E. (2011). Command line analysis of ChIP-seq results. EMBnet.journal 17, https://doi.org/10.14806/ej.17.1.209.

Bhatt, D.M., Pandya-Jones, A., Tong, A.J., Barozzi, I., Lissner, M.M., Natoli, G., Black, D.L., and Smale, S.T. (2012). Transcript dynamics of proinflammatory genes revealed by sequence analysis of subcellular RNA fractions. Cell *150*, 279–290.

Buenrostro, J.D., Giresi, P.G., Zaba, L.C., Chang, H.Y., and Greenleaf, W.J. (2013). Transposition of native chromatin for fast and sensitive epigenomic profiling of open chromatin, DNA-binding proteins and nucleosome position. Nat. Methods *10*, 1213–1218.

Calo, E., and Wysocka, J. (2013). Modification of enhancer chromatin: what, how, and why? Mol. Cell *49*, 825–837.

Chen, G., Shaw, M.H., Kim, Y.G., and Nuñez, G. (2009). NOD-like receptors: role in innate immunity and inflammatory disease. Annu. Rev. Pathol. *4*, 365–398.

ENCODE Project Consortium (2012). An integrated encyclopedia of DNA elements in the human genome. Nature *489*, 57–74.

Daniel, B., Nagy, G., Hah, N., Horvath, A., Czimmerer, Z., Poliska, S., Gyuris, T., Keirsse, J., Gysemans, C., Van Ginderachter, J.A., et al. (2014). The active enhancer network operated by liganded RXR supports angiogenic activity in macrophages. Genes Dev. *28*, 1562–1577.

Elliott, D.E., and Weinstock, J.V. (2012). Helminth-host immunological interactions: prevention and control of immune-mediated diseases. Ann. N Y Acad. Sci. *1247*, 83–96.

Fort, M., Lesley, R., Davidson, N., Menon, S., Brombacher, F., Leach, M., and Rennick, D. (2001). IL-4 exacerbates disease in a Th1 cell transfer model of colitis. J. Immunol. *166*, 2793–2800.

Gandino, L., and Varesio, L. (1990). Immortalization of macrophages from mouse bone marrow and fetal liver. Exp. Cell Res. *188*, 192–198.

Glass, C.K., and Natoli, G. (2016). Molecular control of activation and priming in macrophages. Nat. Immunol. *17*, 26–33.

Glass, C.K., and Saijo, K. (2010). Nuclear receptor transrepression pathways that regulate inflammation in macrophages and T cells. Nat. Rev. Immunol. *10*, 365–376.

Gordon, S., and Martinez, F.O. (2010). Alternative activation of macrophages: mechanism and functions. Immunity *32*, 593–604.

Hah, N., Benner, C., Chong, L.W., Yu, R.T., Downes, M., and Evans, R.M. (2015). Inflammation-sensitive super enhancers form domains of coordinately regulated enhancer RNAs. Proc. Natl. Acad. Sci. USA *112*, E297–E302.

Hoiseth, S.K., and Stocker, B.A. (1981). Aromatic-dependent *Salmonella typhimurium* are non-virulent and effective as live vaccines. Nature *291*, 238–239.

Huang, W., Sherman, B.T., and Lempicki, R.A. (2009). Systematic and integrative analysis of large gene lists using DAVID bioinformatics resources. Nat. Protoc. *4*, 44–57.

Icardi, L., Mori, R., Gesellchen, V., Eyckerman, S., De Cauwer, L., Verhelst, J., Vercauteren, K., Saelens, X., Meuleman, P., Leroux-Roels, G., et al. (2012). The Sin3a repressor complex is a master regulator of STAT transcriptional activity. Proc. Natl. Acad. Sci. USA *109*, 12058–12063.

Johnston, C.J., Robertson, E., Harcus, Y., Grainger, J.R., Coakley, G., Smyth, D.J., McSorley, H.J., and Maizels, R. (2015). Cultivation of *Heligmosomoides polygyrus*: an immunomodulatory nematode parasite and its secreted products. J. Vis. Exp. e52412.

Kang, K., Park, S.H., Chen, J., Qiao, Y., Giannopoulou, E., Berg, K., Hanidu, A., Li, J., Nabozny, G., Kang, K., et al. (2017). Interferon-γ represses M2 gene expression in human macrophages by disassembling enhancers bound by the transcription factor MAF. Immunity *47*, 235–250.e4.

Karagianni, P., and Wong, J. (2007). HDAC3: taking the SMRT-N-CoRrect road to repression. Oncogene *26*, 5439–5449.

Kestler, H.A., Müller, A., Kraus, J.M., Buchholz, M., Gress, T.M., Liu, H., Kane, D.W., Zeeberg, B.R., and Weinstein, J.N. (2008). VennMaster: area-proportional Euler diagrams for functional GO analysis of microarrays. BMC Bioinformatics *9*, 67.

Li, H., and Durbin, R. (2009). Fast and accurate short read alignment with Burrows-Wheeler transform. Bioinformatics *25*, 1754–1760.

Martinez, F.O., Helming, L., Milde, R., Varin, A., Melgert, B.N., Draijer, C., Thomas, B., Fabbri, M., Crawshaw, A., Ho, L.P., et al. (2013). Genetic programs expressed in resting and IL-4 alternatively activated mouse and human macrophages: similarities and differences. Blood *121*, e57–e69.

Mosser, D.M., and Edwards, J.P. (2008). Exploring the full spectrum of macrophage activation. Nat. Rev. Immunol. *8*, 958–969.

Mullican, S.E., Gaddis, C.A., Alenghat, T., Nair, M.G., Giacomin, P.R., Everett, L.J., Feng, D., Steger, D.J., Schug, J., Artis, D., and Lazar, M.A. (2011). Histone deacetylase 3 is an epigenomic brake in macrophage alternative activation. Genes Dev. *25*, 2480–2488.

Murray, P.J., Allen, J.E., Biswas, S.K., Fisher, E.A., Gilroy, D.W., Goerdt, S., Gordon, S., Hamilton, J.A., Ivashkiv, L.B., Lawrence, T., et al. (2014). Macrophage activation and polarization: nomenclature and experimental guidelines. Immunity *41*, 14–20.

Natoli, G., and Andrau, J.C. (2012). Noncoding transcription at enhancers: general principles and functional models. Annu. Rev. Genet. *46*, 1–19.

Nusinzon, I., and Horvath, C.M. (2003). Interferon-stimulated transcription and innate antiviral immunity require deacetylase activity and histone deacetylase 1. Proc. Natl. Acad. Sci. USA *100*, 14742–14747.

Ostuni, R., Piccolo, V., Barozzi, I., Polletti, S., Termanini, A., Bonifacio, S., Curina, A., Prosperini, E., Ghisletti, S., and Natoli, G. (2013). Latent enhancers activated by stimulation in differentiated cells. Cell *152*, 157–171.

Piccolo, V., Curina, A., Genua, M., Ghisletti, S., Simonatto, M., Sabò, A., Amati, B., Ostuni, R., and Natoli, G. (2017). Opposing macrophage polarization programs show extensive epigenomic and transcriptional cross-talk. Nat. Immunol. *18*, 530–540.

Rao, S.S., Huntley, M.H., Durand, N.C., Stamenova, E.K., Bochkov, I.D., Robinson, J.T., Sanborn, A.L., Machol, I., Omer, A.D., Lander, E.S., and Aiden, E.L. (2014). A 3D map of the human genome at kilobase resolution reveals principles of chromatin looping. Cell *159*, 1665–1680.

Rathinam, V.A., and Fitzgerald, K.A. (2016). Inflammasome complexes: emerging mechanisms and effector functions. Cell *165*, 792–800.

Ross-Innes, C.S., Stark, R., Teschendorff, A.E., Holmes, K.A., Ali, H.R., Dunning, M.J., Brown, G.D., Gojis, O., Ellis, I.O., Green, A.R., et al. (2012). Differential oestrogen receptor binding is associated with clinical outcome in breast cancer. Nature *481*, 389–393.

Rückerl, D., Campbell, S.M., Duncan, S., Sutherland, T.E., Jenkins, S.J., Hewitson, J.P., Barr, T.A., Jackson-Jones, L.H., Maizels, R.M., and Allen, J.E. (2017). Macrophage origin limits functional plasticity in helminth-bacterial co-infection. PLoS Pathog. *13*, e1006233.

Ruffell, B., Affara, N.I., and Coussens, L.M. (2012). Differential macrophage programming in the tumor microenvironment. Trends Immunol. *33*, 119–126.

Schmidt, S.F., Larsen, B.D., Loft, A., Nielsen, R., Madsen, J.G., and Mandrup, S. (2015). Acute TNF-induced repression of cell identity genes is mediated by NFκB-directed redistribution of cofactors from super-enhancers. Genome Res. *25*, 1281–1294.

Shen, L., Shao, N., Liu, X., and Nestler, E. (2014). ngs.plot: Quick mining and visualization of next-generation sequencing data by integrating genomic databases. BMC Genomics *15*, 284.

Shlyueva, D., Stampfel, G., and Stark, A. (2014). Transcriptional enhancers: from properties to genome-wide predictions. Nat. Rev. Genet. *15*, 272–286.

Subramanian, A., Tamayo, P., Mootha, V.K., Mukherjee, S., Ebert, B.L., Gillette, M.A., Paulovich, A., Pomeroy, S.L., Golub, T.R., Lander, E.S., and Mesirov, J.P. (2005). Gene set enrichment analysis: a knowledge-based

approach for interpreting genome-wide expression profiles. Proc. Natl. Acad. Sci. USA *102*, 15545–15550.

Takeda, K., Kaisho, T., and Akira, S. (2003). Toll-like receptors. Annu. Rev. Immunol. *21*, 335–376.

Thomas, G.D., Rückerl, D., Maskrey, B.H., Whitfield, P.D., Blaxter, M.L., and Allen, J.E. (2012). The biology of nematode- and IL4Rα-dependent murine macrophage polarization in vivo as defined by RNA-Seq and targeted lipidomics. Blood *120*, e93–e104.

Thorvaldsdóttir, H., Robinson, J.T., and Mesirov, J.P. (2013). Integrative Genomics Viewer (IGV): high-performance genomics data visualization and exploration. Brief. Bioinform. *14*, 178–192.

Trapnell, C., Hendrickson, D.G., Sauvageau, M., Goff, L., Rinn, J.L., and Pachter, L. (2013). Differential analysis of gene regulation at transcript resolution with RNA-seq. Nat. Biotechnol. *31*, 46–53.

Weinstock, J.V., and Elliott, D.E. (2013). Translatability of helminth therapy in inflammatory bowel diseases. Int. J. Parasitol. *43*, 245–251.

Yan, Q., Carmody, R.J., Qu, Z., Ruan, Q., Jager, J., Mullican, S.E., Lazar, M.A., and Chen, Y.H. (2012). Nuclear factor-κB binding motifs specify Toll-like receptor-induced gene repression through an inducible repressosome. Proc. Natl. Acad. Sci. USA *109*, 14140–14145.

Zhang, Y., Liu, T., Meyer, C.A., Eeckhoute, J., Johnson, D.S., Bernstein, B.E., Nusbaum, C., Myers, R.M., Brown, M., Li, W., and Liu, X.S. (2008). Model-based analysis of ChIP-Seq (MACS). Genome Biol. *9*, R137.

STAR★METHODS

KEY RESOURCE TABLE

REAGENT or RESOURCE	SOURCE	IDENTIFIER
Antibodies		
H3K27Ac	Abcam	ab4729; RRID: AB_2118291
P300	Santa Cruz	sc-585; RRID: AB_2231120
PU.1	Santa Cruz	sc-352; RRID: AB_632289
JunB	Santa Cruz	sc-46x; RRID: AB_2130022
IRF8	Santa Cruz	sc-6058; RRID: AB_649510
STAT6	Santa Cruz	sc-981; RRID: AB_632450
C/EBPα	Santa Cruz	sc-61x; RRID: AB_631233
HDAC1	Abcam	ab7028; RRID: AB_305705
HDAC2	Abcam	ab7029; RRID: AB_305706
HDAC3	Abcam	ab7030; RRID: AB_305708
RNA PolII-pS5	Abcam	ab5131; RRID: AB_449369
RNA PolII-pS2	Abcam	ab5095; RRID: AB_304749
p65	Santa Cruz	sc-372; RRID: AB_632037
pro-IL-1β	R&D System	AF401-NA; RRID: AB_416684
ASC	Santa Cruz	sc22514-R; RRID: AB_2174874
NLRP3	AdipoGen	AG-20B-0014; RRID: AB_2490202
pro-caspase-1	AdipoGen	AG-20B-0042; RRID: AB_2490248
NOS2	eBioscience	12/5920; RRID: AB_2572641
Ym1	R&D Systems	BAF2446; RRID: AB_2260451
Bacterial and Virus Strains		
Salmonella enterica enterica serovar Typhimurium strain SL3261	Prof. David Gray	University of Edinburgh
Chemicals, Peptides, and Recombinant Proteins		
mouse recombinant IL-4	Peprotech	214-14
LPS (*Salmonella enterica* serotype minnesota Re 595)	Sigma Aldrich	L6261
Critical Commercial Assays		
TruSeq RNA Sample Prep Kit v2 -Set A (48rxn)	Illumina	RS-122-2001
Ovation Ultralow System v2 1-16 (32rxn) without magnetic beads	Nugen	0344NB-32
TruSeq ChIP SamplePreparation Kit - Set A	Illumina	IP-202-1012
Nextera DNA Sample Preparation Kit (24 Samples)	Illumina	FC-121-1030
NEBNext Multiplex Small RNA Prep Set for Illumina (Set1)	Illumina	E7300 S
IL-1β ELISA kit	R&D System	DY401-05
FLICA 660 far-red fluorescence Caspase-1 Assay Kit	ImmunoChemistry Technologies	9122
Deposited Data		
RNA-seq, ChIP-seq, GRO-seq and ATAC-seq	This paper	GEO: GSE106706
Microarray data from WT and HDAC3 KO BMDMs	(Mullican et al., 2011)	GEO: GSE33609
SMRT and NCoR ChIP-seq	(Barish et al., 2012)	GEO: GSE27060
H3K4m1 ChIP-seq	(Ostuni et al., 2013)	GEO: GSE38379

(*Continued on next page*)

Continued

REAGENT or RESOURCE	SOURCE	IDENTIFIER
CTCF and RAD21 ChIP-seq	(Daniel et al., 2014)	SRA: SRP019970
RNA-seq data from Thio-Mac and Ne-Mac	(Thomas et al., 2012)	ArrayExpress: E-MTAB-995
Experimental Models: Cell Lines		
primary bone marrow-derived macrophages	WT C57BL/6 and *Stat6*$^{-/-}$ mice	N/A
immortalized bone marrow-derived macrophages	WT C57BL/6 and *Ncor1*$^{fl/fl}$*Lys2 Cre* mice	N/A
Experimental Models: Organisms/Strains		
C57BL/6	The Jackson Laboratory	N/A
Stat6$^{-/-}$	The Jackson Laboratory	N/A
Ncor1$^{fl/fl}$	Prof. Johan Auwerx	N/A
Oligonucleotides		
Primers for mRNA expression	This paper	see Table S6
Primers for eRNA expression	This paper	see Table S6
Primers for ChIP-qPCR experiments	This paper	see Table S6
Software and Algorithms		
GraphPad Prism	GraphPad Software, Inc	https://www.graphpad.com/
Ingenuity Pathway Analysis	QIAGEN	https://www.qiagenbioinformatics.com/products/ingenuity-pathway-analysis/
TopHat	(Trapnell et al., 2013)	http://cole-trapnell-lab.github.io/projects/tophat/
Cufflinks	(Trapnell et al., 2013)	http://cole-trapnell-lab.github.io/projects/cufflinks/
Gene Set Enrichment Analysis (GSEA)	(Subramanian et al., 2005)	http://software.broadinstitute.org/gsea/index.jsp
DAVID 6.8	(Huang et al., 2009)	https://david.ncifcrf.gov/tools.jsp
Burrows-Wheeler Alignment Tool	(Li and Durbin, 2009)	http://bio-bwa.sourceforge.net/
MACS2	(Zhang et al., 2008)	https://github.com/taoliu/MACS
DiffBind v2.0.5	(Ross-Innes et al., 2012)	https://bioconductor.org/packages/release/bioc/html/DiffBind.html
VennMaster	(Kestler et al., 2008)	http://sysbio.uni-ulm.de/?Software:VennMaster
IGV2.3	(Thorvaldsdóttir et al., 2013)	http://software.broadinstitute.org/software/igv/igv2.3
ngs.plot	(Shen et al., 2014)	https://github.com/shenlab-sinai/ngsplot
Other		
Heligmosomoides polygyrus	Prof. Richard Grencis	University of Manchester

CONTACT FOR REAGENT AND RESOURCE SHARING

Further information and requests for resources and reagents should be directed to and will be fulfilled by the Lead Contact, Laszlo Nagy (lnagy@sbpdiscovery.org).

EXPERIMENTAL MODEL AND SUBJECT DETAILS

Mice

All strains are on C57BL/6 genetic background. *Stat6*$^{-/-}$ is a full body knockout and it was purchased from The Jackson Laboratory. Animals were housed under minimal disease conditions and the experiments were carried out under institutional ethical guidelines and licenses.

Bone Marrow-Derived Macrophages

Isolation and differentiation were completed as described earlier (Daniel et al., 2014). Isolated bone marrow-derived cells were differentiated for 6 days in the presence of L929 supernatant. Differentiated BMDMs were treated with IL-4 (20 ng/ml), LPS (100 nM) and ATP (5mM) for the indicated period of time.

Immortalization of mouse bone marrow-derived macrophages

Bone marrow-derived cells were immortalized using the J2 cell line continuously producing the J2 virus encoding v-raf and v-myc oncogenes (Gandino and Varesio, 1990). J2 cells were grown in DMEM containing 20% FBS. Bone marrow cells were seeded in immortalization media I. (90% J2 supernatant, 5% HyClone FBS, 10ug/ml Polybrene 0.1%, L929 supernatant 5%) and incubated overnight. On the second day supernatant was collected and spun down to pellet floating cells. Adherent cells were scraped and re-plated in a new Petri dish using immortalization media II. (20% J2 supernatant, 10% HyClone FBS, 10ug/ml Polybrene 0.1%, L929 supernatant 10%, 60% DMEM) and incubated for 6 days. After the immortalization cells were kept in regular macrophage differentiation media (20% FBS, 30% L929 supernatant and 50% DMEM containing 1% antibiotics).

METHOD DETAILS

RNA-seq

cDNA library for RNA-Seq was generated from 1 μg total RNA using TruSeq RNA Sample Preparation Kit (Illumina, San Diego, CA, USA) according to the manufacturer's protocol. Briefly, poly-A tailed RNAs were purified by oligodT conjugated magnetic beads and fragmented on 94 C degree for 8 minutes, then 1st strand cDNA was transcribed using random primers and SuperScript II reverse transcriptase (Lifetechnologies, Carslbad, CA, USA). Following this step second strand cDNA synthesized, double stranded cDNA end repaired and 3′ ends adenylated then Illumina index adapters were ligated. After adaptor ligation enrichment PCR was performed to amplify adaptor ligated cDNA fragments. Fragment size distribution and molarity of libraries were checked on Agilent BioAnalyzer DNA1000 chip (Agilent Technologies, Santa Clara, CA, USA). Paired read 100bp sequencing runs were performed on Illumina HiScan SQ instrument (Illumina, San Diego, CA, USA).

ChIP-seq and ChIP-qPCR

ChIP was performed essentially as previously described (Daniel et al., 2014). Libraries were prepared either with Ovation Ultralow Library Systems (Nugen) or TruSeq ChIP library systems (Illumina) according to the manufacturer's instructions. The following antibodies were used: H3K27Ac (ab4729), P300 (sc-585), PU.1 (sc-352), JunB (sc-46x), IRF8 (sc-32528x), STAT6 (sc-981), C/EBPα (sc-61X), HDAC1 (ab7028), HDAC2 (ab7029), HDAC3 (ab4729), RNA PolII-pS5 (ab5131) and RNA PolII-pS2 (ab5095), p65 (sc-372). Primer sequences for ChIP-qPCR are available in Table S6.

ATAC-seq

ATAC-seq was carried out as described earlier with minor modification (Buenrostro et al., 2013). Cells were scraped and counted to achieve 50k/ml in ice-cold PBS. Cell suspension was further diluted to 25k/ml and nuclei were isolated with ATAC-LB (10mM Tris-HCl pH7.4, 10mM NaCl, 3mM MgCl2, 0.1% IGEPAL). Nuclei from 25k cells were used for tagmentation using Nextera DNA Library Preparation Kit (Illumina) from two biological replicates. After tagmentation DNA was purified with Minelute PCR Purification Kit (QIAGEN). Tagmented DNA was amplified with Kapa Hifi Hot Start Kit (Kapa Biosystems) using 9 PCR cycle. Amplified libraries were purified again with Minelute PCR Purification Kit. Fragment distribution of libraries was assessed with Agilent Bioanalyzer and libraries were sequenced on a HiSeq 2500 platform.

GRO-seq

GRO-seq was performed as described earlier (Daniel et al., 2014), but the libraries were prepared with NEBNext Small RNA Library Prep set for Illumina.

Real-Time Quantitative PCR for enhancer RNA and mRNA detection (qPCR)

RNA was isolated with Trizol reagent (Ambion). RNA was reverse transcribed with High-Capacity cDNA Reverse Transcription Kit (Applied Biosystems) according to manufacturer's protocol. Transcript quantification was performed by qPCR reactions using SYBR green master mix (BioRad). Transcript levels were normalized to *Ppia*. Primer sequences are available in Table S6.

LDH release

LDH activity was measured in the supernatants of unstimulated and IL-4-pretreated WT and STAT6KO bone marrow-derived macrophages after IL-4 pretreatment and/or LPS/ATP costimulation (LPS-exposed BMDMs were treated with ATP for 30 min) by commercially available LDH UV assay on Cobas c 501 instrument (Roche Diagnostics, Mannheim, Germany). This measurement is based on the conversion of L-lactate to pyruvate along with the reduction of NAD+ to NADH. The initial rate of the NADH formation was directly proportional to the catalytic LDH activity determined by photometrically measuring the absorbance increment at 340 nm.

Measurement of IL-1β production

LPS-exposed BMDMs were treated with ATP for 45 min. Supernatants from ATP-treated macrophages were collected, centrifuged and stored at −20°C until further use. IL-1β was measured from samples using ELISA kit (DY401-05, R&D System) according to the manufacturer's instructions and analyzed on FlexStation 3 Microplate Reader (Molecular Devices). The minimum detectable dose is 15.6 pg/ml.

Western Blot analysis

Cells were harvested and centrifuged, then they were lysed in loading buffer (62,5mM Tris pH = 8.8, 25% glycerol, 2% SDS, 1% β-mercaptoethanol and 1% BPB). Before loading all samples were boiled for 10 minutes. Proteins were separated by SDS-PAGE and transferred onto nitrocellulose membranes. Membranes were then blocked with 5% non-fat milk, washed briefly, incubated with primary antibodies at 4°C overnight. Pro-IL-1β (AF401-NA) was from R&D System, ASC (sc22514-R) was from Santa Cruz, pro-caspase-1 (AG-20B-0042) and NLRP3 (AG-20B-0014) antibodies were obtained from AdipoGen. Primary antibodies were incubated with corresponding horseradish peroxidase-conjugated secondary antibodies from BioRad for 1 hour at room temperature. Proteins were visualized by Supersignal West-Pico peroxide/luminol enhancer solution from Pierce. To verify the loading of equal amount of protein sample, the β-actin (Sigma-Aldrich) expression was detected.

Laser Scanning Cytometry

Caspase-1 activity and pyroptotic cell death by propidium iodine staining was measured in single cells using imaging Laser Scanning Cytometry (LSC). Mouse macrophages were cultured, treated, stained and imaged in 8 well IBIDI (Martinsried, Germany) slides with an initial concentration of 15,000 cells per well. Sub-vital staining was performed in culture medium at room temperature for 20 minutes by Hoechst 34580 (10 microg/ml), propidium iodine (10 microg/ml), Alexa 488 tagged Annexin V (1 microg/ml) and caspase-1 specific FLICA® 660 (FLICA® 660 far-red fluorescence Caspase-1 Assay Kit was used according to the description of manufacturer; ImmunoChemistry Technologies, LLC). In some experiments specific Caspase-1/ICE Inhibitor Z-WEHD-FMK (R&D Systems, Inc. Minneapolis, MN, USA) was also used before FLICA labeling. For LSC imaging an iCys Research Cytometer (formerly CompuCyte; Thorlabs Imaging Systems, Sterling, VA) was used with its iNovator Application Development Toolkit software. Hoechst, Alexa, PI and FLICA fluorescence dyes were excited separately with 405, 488, 488, 633 nm laser lines and detected at 430-470, 515-545, 650-700, 650 and above nanometers, respectively. Single cell data were gated according to their area, DNA content and nuclear shape and fluorescence pixel integral, maximum pixel intensity and average pixel intensity parameters with raw images were recorded for all dyes. For cytoplasmic caspase-1 activity measurements dynamic background subtraction was applied. Gated single cell FCS data were exported from LSC software and contour plots were generated in FCS Express 5 flow and image cytometry data analysis software (*De Novo* Software, Glendale, CA, USA).

In vivo infection model

Heligmosomoides polygyrus life cycle was maintained in house and infective thirdstage larvae (L3) were obtained as described elsewhere (Johnston et al., 2015). Mice were infected with 200 *H polygyrus* L3 by oral gavage. The attenuated, aroA deficient *Salmonella enterica enterica* serovar Typhimurium strain SL3261 (Hoiseth and Stocker, 1981) was cultured as stationary overnight culture from frozen stock in Luria-Bertani broth. Co-infections were carried out as described previously (Rückerl et al., 2017). Briefly, animals were injected i.p. with ~1x10^6 CFU *Salmonella* Typhimurium diluted in PBS or received 1 mg/kg LPS from *Salmonella enterica* ser. Minnesota (Sigma Aldrich L4641) 9 days after *H.polygyrus* infection. 6h after bacterial inoculation peritoneal macrophages were isolated by lavage, purified by adherence for 2 h to cell culture plastic and total RNA extracted.

Flow cytometry

All antibodies were purchased from Biolegend UK unless otherwise indicated. Equal numbers of cells were stained with LIVE/DEAD cell viability assay (Life Technologies) and blocked with 5 μg/mL anti CD16/32 (2.4G2, BD Biosciences) and heatinactivated normal mouse serum (1:10) in FACS buffer (0.5% BSA and 2 mM EDTA in Dulbecco's PBS) before surface staining with antibodies to F4/80 (BM8), SiglecF (E502440), Ly6C (HK1.4), Ly-6G (1A8), TCRβ (H57-597), CD11b (M1/70), CD11c (N418), I-A/I-E (M5/114.15.2), CD19 (6D5) and CD115 (AFS98). Detection of intracellular Ym1 and NOS2 was performed directly *ex vivo*. Cells were stained for surface markers then fixed with 2% paraformaldehyde (Sigma Aldrich), permeabilized using Permeabilization Buffer (eBioscience) and stained with directly labeled antibodies to NOS2 (CXNFT; eBioscience) or biotinylated polyclonal goat antiYm1 (R&D Systems) followed by streptavidin-PerCP (Biolegend). Expression of Ym1 and NOS2 was determined relative to appropriate polyclonal or monoclonal isotype controls.

Samples were acquired on a BD LSR II using BD FACSDiva software (BD Bioscience) and post-acquisition analysis performed using FlowJo v9 software (Tree Star Inc.). Macrophages were identified as lineage negative (CD19-,TCRb-,Ly6G-,SiglecF-), CD11b+ CD115+.

QUANTIFICATION AND STATISTICAL ANALYSIS

RNA-seq analysis

TopHat and Cufflinks toolkits (Trapnell et al., 2013) were used for mapping spliced reads to the mm10 mouse assembly with default parameters, making transcript assemblies, and getting and sorting gene expression data. Genes with at least 1 FPKM (Fragments Per Kilobase per Million mapped reads) expression value in at least one sample were considered to be expressed. In the downstream analysis 2-way anova and post hoc tests were performed on WT and *Stat6*$^{-/-}$ macrophages exposed to IL-4 for 1, 3, 6 and 24 hours in R using the aov() and TukeyHSD() functions of the MASS package. Differences were considered statistically significant at p value < 0.05 and FC > 2. For IL-4 pretreatment-LPS datasets, LPS-induced genes were considered statistically significant at p value < 0.05 compared to the control and then these genes were clustered based on their sensitivity (p value < 0.05) to IL-4 pretreatment as follows: attenuated response - Cluster 1; insensitive - Cluster 2; increased response - Cluster 3. K-means clustering was performed in R using the function kmeans from package stats. Gene Set Enrichment Analysis was done by GSEA v2.2.0 (Subramanian et al., 2005). KEGG pathway enrichment analyses were done using the DAVID web application (Huang et al., 2009). Heatmaps were drawn using the R package pheatmap.

ATAC-seq, ChIP-seq and GRO-seq analyses

The primary analysis of the ATAC-seq, ChIP-seq and GRO-seq raw sequence reads was carried out using our ChIP-seq analysis command line pipeline (Barta, 2011). Briefly, Burrows-Wheeler Alignment Tool (BWA, (Li and Durbin, 2009)) was used to align the reads to mm10 genome assembly with default parameters. MACS2 (Zhang et al., 2008) (with '-B' and '-SPMR' options) was used for predicting transcription factor peaks and nucleosome free regions (q-value $\leq$ 0.01), and findPeaks.pl (with '-size 1000', '-minDist 2500' and '-style histone' options) for histone regions. Artifacts were removed using the ENCODE blacklist (ENCODE Project Consortium, 2012). Predicted peaks were sorted by average coverage (RPKM, Reads Per Kilobase per Million mapped reads). Average coverage of the predicted peaks and significantly changing regions (p value $\leq$ 0.05) were calculated by DiffBind v2.0.5 (Ross-Innes et al., 2012). Intersections, subtractions and merging of the predicted peaks were made with BedTools. Proportional Venn diagrams were generated with VennMaster (Kestler et al., 2008). Genome coverage files (bedgraph files) for visualization were generated by makeUCSCfile.pl (HOMER) and then converted into tdf files using igvtools with 'toTDF' option. *De novo* motif discovery was performed on the 100 bp vicinity of the peak summits using findMotifsGenome.pl with options '–len "10,12,14,16"' and '-size 200' on the repeat-masked mouse genome (mm10r) from HOMER. The HOMER option '-style groseq' was used for GRO-seq samples. Integrative Genomics Viewer (IGV2.3, Broad Institute) was used for data browsing (Thorvaldsdóttir et al., 2013) and creating representative snapshots. Normalized tag counts for Meta histograms and RD plots were generated by annotatePeaks.pl from HOMER (with option '-hist 10' for histograms and with options '-ghist' and '-hist 10' for RD plots) and visualized by R using package ggplot2 or by Java TreeView, respectively. Gene body metaplots were created using ngs.plot software (Shen et al., 2014). Pearson's correlation coefficients between GRO-seq, PolII S2, PolII S5 and H3K27ac ChIP-seq data (fold change of RPKM values upon 1h IL-4 treatment on the merged replicates using a custom bash script) were calculated in R using function cor() from package stats. Changes on boxplots were considered significant at p < 0.00001 using paired t test and the average of fold differences at the individual enhancers $\geq$ 1.15.

Domain prediction

ChIP-seq raw reads of 47 CTCF and 42 Cohesin (RAD21, SMC1/3 or SA1/2) samples were downloaded from the Sequence Read Archive of NCBI and processed using our ChIP-seq analysis command line pipeline (Barta, 2011). Consensus CTCF peak summits were defined as the average genomic location of at least two summits within 51 bp. Consensus peak summits for Cohesin were defined in the same manner. Insulator peak summits were determined from those consensus CTCF peak summits that were closer to a consensus Cohesin peak summit than 51 bp. Motif enrichments were calculated in two rounds by findMotifsGenome.pl (HOMER) from the 100 bp region around the 5000 most ubiquitous insulator peak summits. Having mapped the putative elements matching with the CTCF motif of the first search by annotatePeaks.pl (HOMER), we used those top 5000 regions that lacked these hits. Score 6 was set as a threshold for both CTCF motif matrices, and to filter putative CTCF elements in the case of multiple occurrences at the same region those hits were preferred that followed the direction of the CTCF/Cohesin peak location compared to each other (Rao et al., 2014) and had the highest motif score. Insulators showing clear protein-binding direction without predicted element were also included in domain prediction. Average coverage (RPKM) of CTCF and RAD21 ChIP-seq derived from bone marrow-derived macrophages was calculated on the 100 bp region around insulators, and those regions were filtered out that had an RPKM value exceeding the hundred-thousandth of the summed density of all regions per sample in both samples. The closest insulators showing convergent direction within 1Mb distance but farther than 1kb were assigned to each other and called domains if their coverage showed less than 2-fold difference for both proteins. In the case of overlapping domains, those were filtered having the highest insulator coverage. "Negative" domains with divergent insulators were defined between the final convergent domains. Association scores between STAT6-bound enhancers and IL-4-regulated gene clusters were calculated and visualized by package pheatmap using option scale = "column" (scaling by RNA-seq clusters).

Statistical analysis

Statistical analysis for qRT-PCR, ChIP-qPCR, ELISA, LDH-release assay, FACS analysis and densitometry analysis of western blot: the error bars represent standard deviation (SD). The two-tailed Student's t test was used to evaluate the significance of differences between two groups. Quantification and alignments of NGS analysis for RNA-seq, ChIP-seq and ATAC-seq are also described in more detail in the methods section above.

DATA AND SOFTWARE AVAILABILITY

The accession number for the RNA-seq, ChIP-seq, GRO-seq, and ATAC-seq data reported in this paper is GEO: GSE106706. Microarray and RNA-seq datasets were downloaded from NCBI GEO depository (GSE33609) and ArrayExpress (E-MTAB-995). ChIP-seq datasets were downloaded from NCBI GEO depository (GSE27060 and GSE38379) as well as Sequence Read Archive (SRP019970). The used genome-wide datasets are collected in Table S7.

Immunity, Volume *48*

Supplemental Information

The Transcription Factor STAT6 Mediates Direct Repression of Inflammatory Enhancers and Limits Activation of Alternatively Polarized Macrophages

Zsolt Czimmerer, Bence Daniel, Attila Horvath, Dominik Rückerl, Gergely Nagy, Mate Kiss, Matthew Peloquin, Marietta M. Budai, Ixchelt Cuaranta-Monroy, Zoltan Simandi, Laszlo Steiner, Bela Nagy Jr., Szilard Poliska, Csaba Banko, Zsolt Bacso, Ira G. Schulman, Sascha Sauer, Jean-Francois Deleuze, Judith E. Allen, Szilvia Benko, and Laszlo Nagy

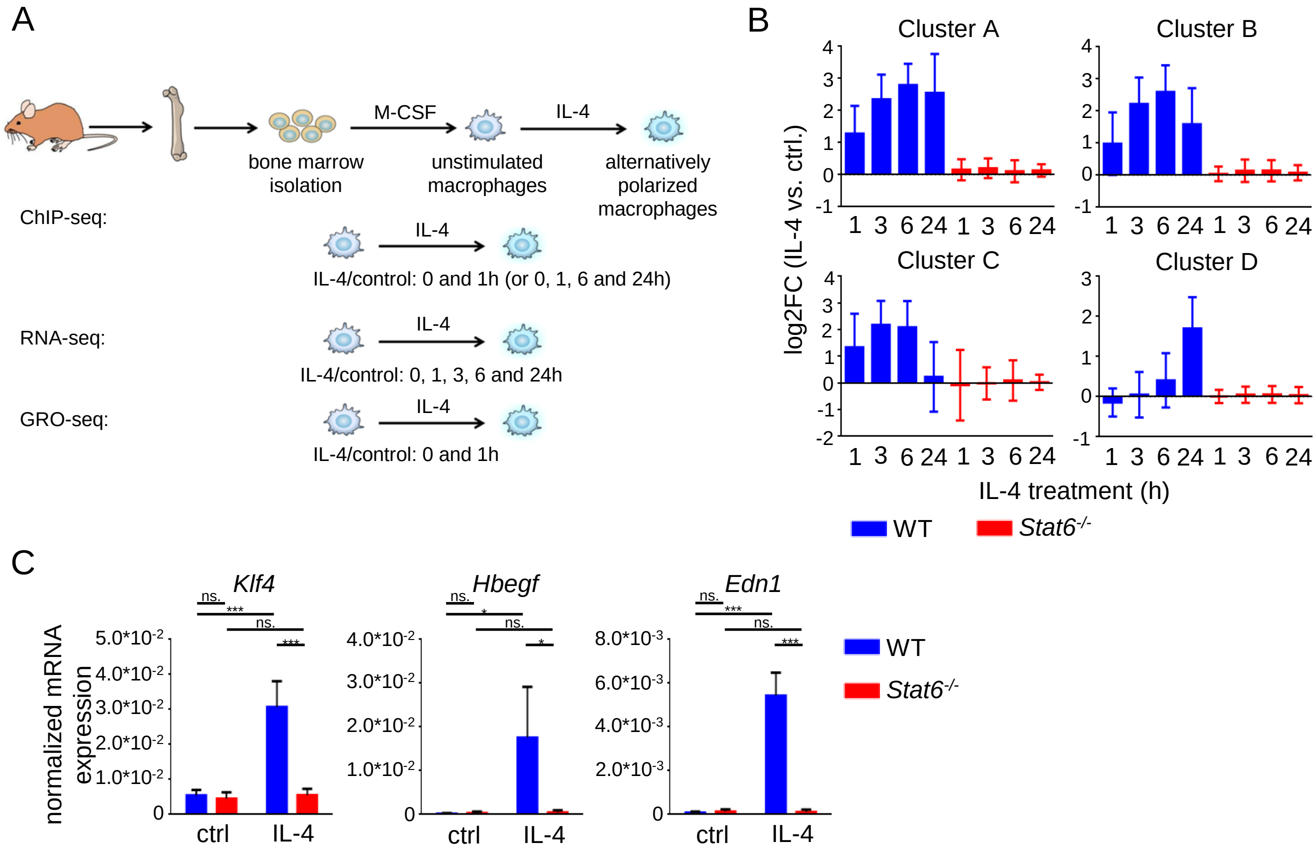

Figure S1. (Related to Figure 1) Identification of IL-4-STAT6 signaling pathway-mediated global transcriptional changes in mouse BMDMs. (A) Schematic representation of applied mouse alternative macrophage polarization protocol and experimental system. (B) The average fold change from the IL-4-repressed gene cluster at the indicated time points following IL-4 stimulation in WT (n=4) and *Stat6*$^{-/-}$ (n=2) BMDMs. Error bars represent means ±SD. (C) RT-qPCR analysis of gene expression on a set of IL-4-activated genes in WT and Stat6$^{-/-}$ BMDMs. BMDMs were treated with IL-4 for 6 hours. Data are representative of five individual animals per genotype from two independent experiments. **P*<0.05, ***P*<0.01, ****P*<0.001, ns. indicates not significant change. Error bars represent means ±SD.

A

	RNAPII-pS2⁺ gene bodies
All	18034
IL-4-repressed	3008
IL-4-induced	2923

B

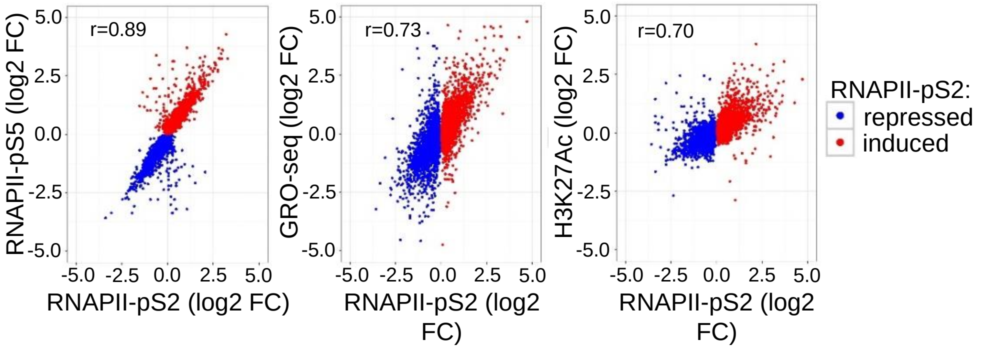

C

RNAPII-pS2 positive gene bodies

Gene expression clusters (Figure 1A)	IL-4-repressed	IL-4-induced
A (32)	0	31
B (164)	1	142
C (292)	3	235
D (501)	125	137
E (625)	406	18

D

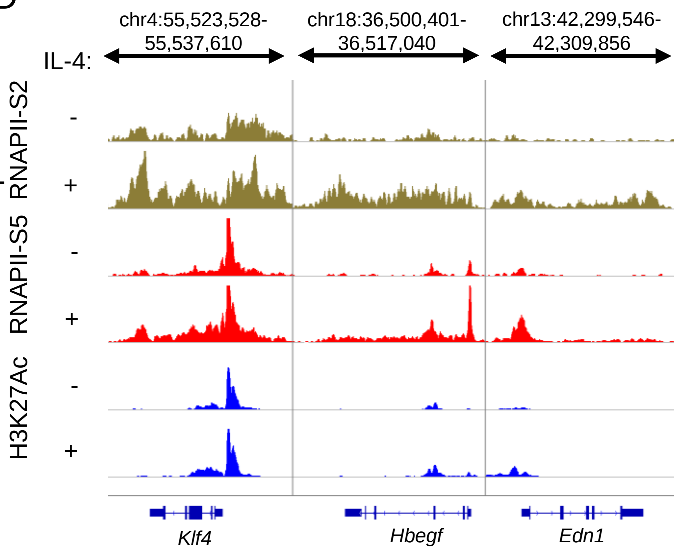

Figure S2. (Related to Figure 2) Characterization of RNAPII binding, nascent RNA expression and H3K27Ac enrichment at the gene bodies of IL-4-responsive genes. (A) The number of gene bodies associated with IL-4-dependent regulation of RNAPII-pS2 binding ($p \leq 0.1$) in BMDMs. (B) Correlation of RNAPII-pS2 binding with RNAPII-pS5 binding, nascent RNA expression as well as H3K27Ac enrichment at the IL-4-regulated RNAPII-pS2 binding-associated gene bodies. Data (H3K27Ac, RNAPII-pS2 and RNAPII-pS5) are combined from two independent biological replicates. (C) Overlap between IL-4-regulated gene expression clusters (Figure 1A) and IL-4-regulated RNAPII-pS2-associated gene bodies (Figure S2A). (D) H3K27Ac, RNAPII-pS5 and RNAPII-pS2 ChIP-Seq signals at the selected IL-4-activated gene bodies. ChIP-seq signals are visualized by the Integrative Genomics Viewer. Data are representative of two independent biological replicates.

BMDMs were treated with IL-4 for 1 hour in (A, B and D).

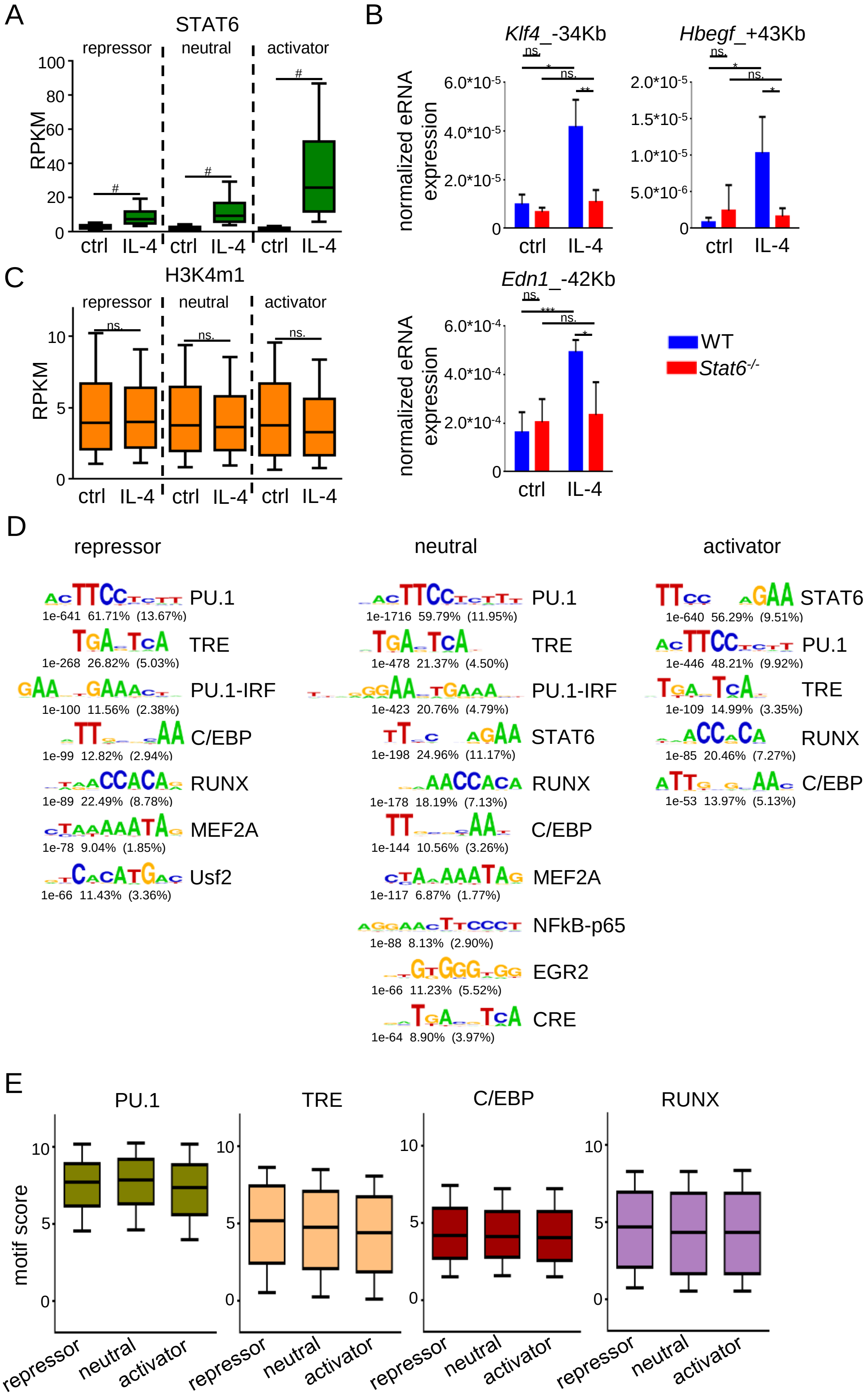
A
STAT6
repressor
neutral
activator
RPKM
ctrl
IL-4
B
Klf4_-34Kb
Hbegf_+43Kb
Edn1_-42Kb
normalized eRNA expression
WT
Stat6-/-
C
H3K4m1
D
PU.1
TRE
PU.1-IRF
C/EBP
RUNX
MEF2A
Usf2
STAT6
NFkB-p65
EGR2
CRE
1e-641 61.71% (13.67%)
1e-268 26.82% (5.03%)
1e-100 11.56% (2.38%)
1e-99 12.82% (2.94%)
1e-89 22.49% (8.78%)
1e-78 9.04% (1.85%)
1e-66 11.43% (3.36%)
1e-1716 59.79% (11.95%)
1e-478 21.37% (4.50%)
1e-423 20.76% (4.79%)
1e-198 24.96% (11.17%)
1e-178 18.19% (7.13%)
1e-144 10.56% (3.26%)
1e-117 6.87% (1.77%)
1e-88 8.13% (2.90%)
1e-66 11.23% (5.52%)
1e-64 8.90% (3.97%)
1e-640 56.29% (9.51%)
1e-446 48.21% (9.92%)
1e-109 14.99% (3.35%)
1e-85 20.46% (7.27%)
1e-53 13.97% (5.13%)
E
motif score

Figure S3. (Related to Figure 3) Characterization of RNAPII-pS5 positive STAT6 peak clusters. (A) Box plot representation of STAT6 transcription factor binding at the STAT6-occupied RNAPII-pS5$^+$ genomic regions in WT macrophages. BMDMs were treated with IL-4 for 1 hour. Boxes encompass the 25th to 75th percentile RPKMs. Whiskers extend to the 10th and 90th percentiles. Changes were considered significant at $p<0.00001$ using paired t-test and the average of fold differences at the individual enhancers ≥1.15. # $p<0.00001$ and average fold difference≥1.15, ns. indicates not significant. (B) RT-qPCR measurements of eRNA expression at IL-4-repressed enhancers in WT and *Stat6*$^{-/-}$ macrophages. BMDMs were treated with IL-4 for 1 hour. Data are representative of five individual animals per genotype from two independent experiments. **P*<0.05, ***P*<0.01, ****P*<0.001, ns. indicates not significant change. Error bars represent means ±SD. (C) Box plot representation of H3K4m1 enrichment at the surrounding genomic regions of the identified STAT6 peak clusters in WT macrophages. Macrophages were treated with IL-4 for 4 hours. Boxes encompass the 25th to 75th percentile RPKMs. Whiskers extend to the 10th and 90th percentiles. Changes were considered significant at $p<0.00001$ using paired t-test and the average of fold differences at the individual enhancers≥1.15. # $p<0.00001$ and average fold difference≥1.15, ns. indicates not significant. (D) De novo motif enrichment identification under different STAT6 peak clusters from ChIP-seq data using HOMER. "Target %" refers to the ratio of the peaks having the given motif, and "Bg %" refers to the ratio of a random background. (E) Box plot representation of PU.1, TRE, C/EBP and RUNX motif scores on genomic regions under different STAT6 peak clusters. Boxes encompass the 25th to 75th percentile motif scores. Whiskers extend to the 10th and 90th percentiles.

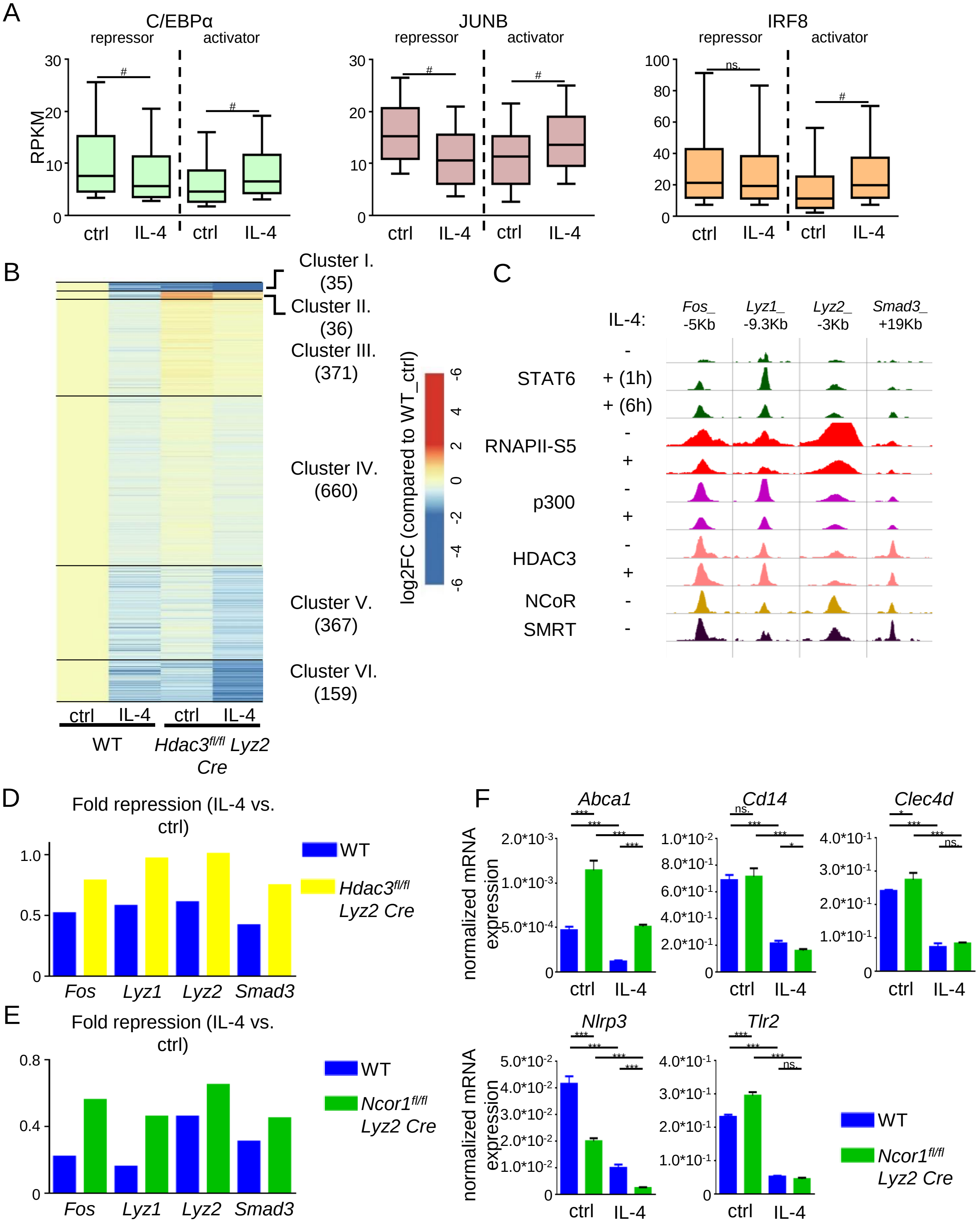
A
C/EBPα
JUNB
IRF8
repressor
activator
RPKM
ctrl
IL-4
#
ns.
B
Cluster I. (35)
Cluster II. (36)
Cluster III. (371)
Cluster IV. (660)
Cluster V. (367)
Cluster VI. (159)
WT
Hdac3fl/fl Lyz2 Cre
log2FC (compared to WT_ctrl)
C
IL-4:
Fos_ -5Kb
Lyz1_ -9.3Kb
Lyz2_ -3Kb
Smad3_ +19Kb
STAT6
+ (1h)
+ (6h)
RNAPII-S5
p300
HDAC3
NCoR
SMRT
D
Fold repression (IL-4 vs. ctrl)
WT
Hdac3fl/fl Lyz2 Cre
Fos
Lyz1
Lyz2
Smad3
E
Fold repression (IL-4 vs. ctrl)
WT
Ncor1fl/fl Lyz2 Cre
F
Abca1
Cd14
Clec4d
Nlrp3
Tlr2
normalized mRNA expression
WT
Ncor1fl/fl Lyz2 Cre

Figure S4. (Related to Figure 4) Characterization of IL-4-STAT6 signaling pathway-mediated transcriptional re-ression and activation at the STAT6 bound enhancers. (A) Box plot representation of JunB, C/EBPα and IRF8 ChIP-seq signals at the STAT6-occupied genomic regions in WT macrophages. BMDMs were treated with IL-4 for 1 hour. Boxes encompass the 25th to 75th percentile RPKMs. Whiskers extend to the 10th and 90th percentiles. Changes were considered significant at $p<0.00001$ using paired t-test and the average of fold differences at the individual enhancers≥1.15. # $p<0.00001$ and average fold difference≥1.15, ns. indicates not significant. (B) Heat map representation of IL-4-repressed gene clusters ($p\leq0.05$) in unstimulated and IL-4 stimulated WT and *Hdac3$^{fl/fl}$ Lyz2 Cre* murine macrophages. Clustering was based on the participation of HDAC3 in the IL-4-mediated repression. BMDMs were treated with IL-4 for 24 hours. (C) Representative examples of IL-4-STAT6-HDAC3-repressed genes-associated enhancers. Genome browser view of the merge of STAT6, RNAPII-pS5, p300 and HDAC3-specific ChIP-seq from unstimulated or IL-4-stimulated WT as well as SMRT and NCoR ChIP-seq from unstimulated WT macrophages. (D) Fold repression of *Fos*, *Lyz1*, *Lyz2* and *Smad3* expression between IL-4-stumulated and unstimulated WT as well as *Hdac3$^{fl/fl}$ Lyz2 Cre* BMDMs. BMDMs were treated with IL-4 for 24 hours. (E) Fold repression of *Fos*, *Lyz1*, *Lyz2* and *Smad3* expression between IL-4-stumulated and unstimulated WT as well as *Ncor1$^{fl/fl}$ Lyz2 Cre* iBMDMs. BMDMs were treated with IL-4 for 24 hours. (F) RT-qPCR-based measurement of *Abca1*, *Cd14*, *Clec4d*, *Nlrp3* and *Tlr2* expression in unstimulated as well as IL-4 stimulated WT and *Ncor1$^{fl/f}$ Lyz2 Cre* iBMDMs. BMDMs were treated with IL-4 for 24 hours. Data represent the mean and SD of three independent biological replicates. *$P<0.05$, **$P<0.01$, ***$P<0.001$, ns. indicates no significant difference.

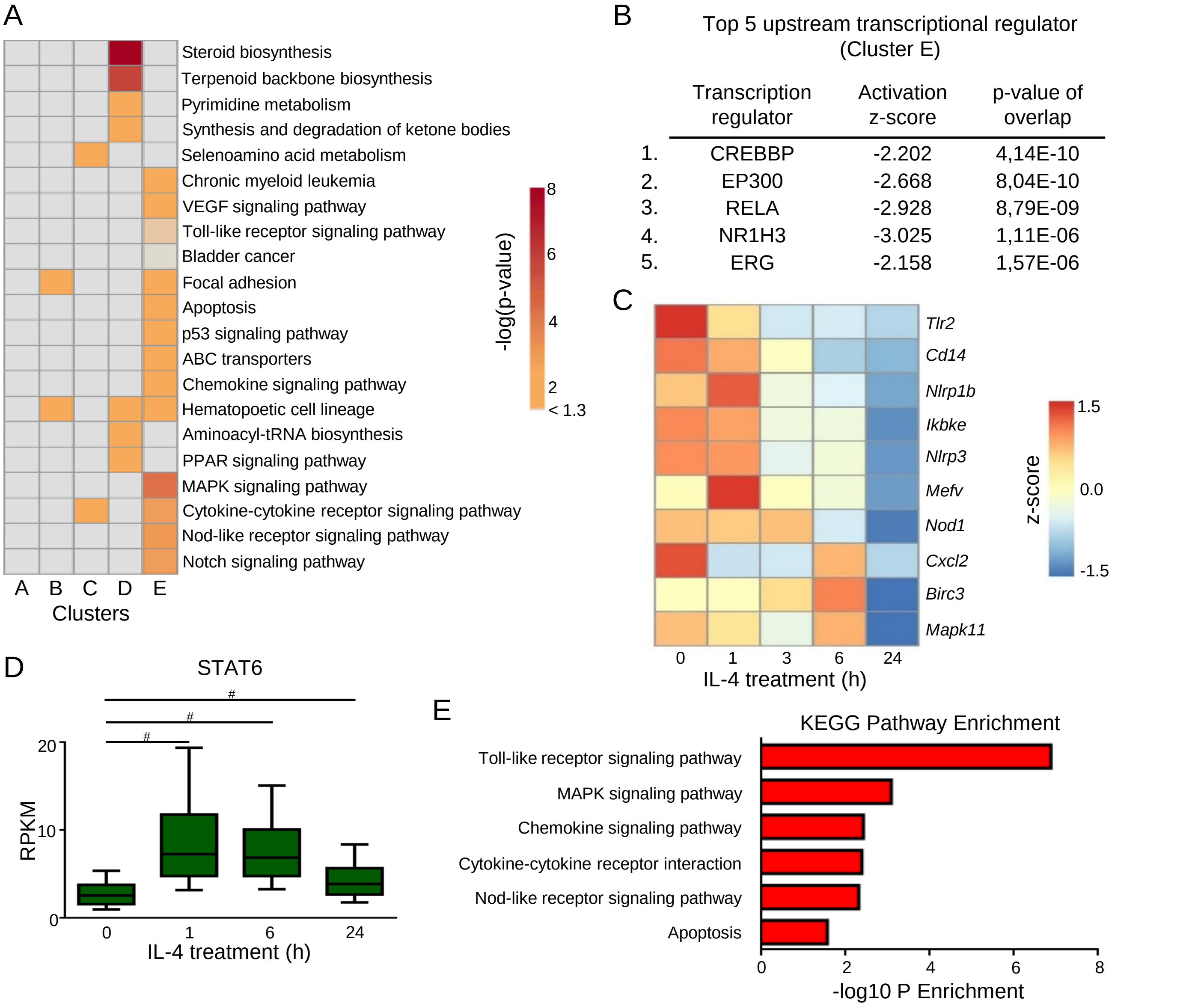

Figure S5. (Related to Figure 5) (A) Five IL-4-regulated gene clusters-associated significantly enriched KEGG pathway categories. (B) Ingenuity Pathway Analysis algorithm-based prediction of top upstream transcriptional regulators of IL-4-repressed genes. Transcription regulators showing *p*-value overlap <0.01 and regulation *z*-score >2 or <-2 are shown. (C) Heat map representation of IL-4-regulated gene expression of the selected members of Toll-like and Nod-like receptor pathways in murine BMDMs. BMDMs were treated with IL-4 for 1, 3, 6 and 24 hours. (D) Box plot representation of STAT6 transcription factor binding at the "repressor" STAT6-occupied RNAPII-pS5^{+} genomic regions in WT BMDMs. BMDMs were treated with IL-4 for 1, 6 and 24 hours. Boxes encompass the 25th to 75th percentile RPKMs. Whiskers extend to the 10th and 90th percentiles. Changes were considered significant at p<0.00001 using paired t-test and the average of fold differences at the individual enhancers ≥1.15. # p<0.00001 and average fold difference≥1.15, ns. indicates not significant. (E) IL-4-attenuated LPS-inducible gene cluster-associated significantly enriched KEGG pathway categories.

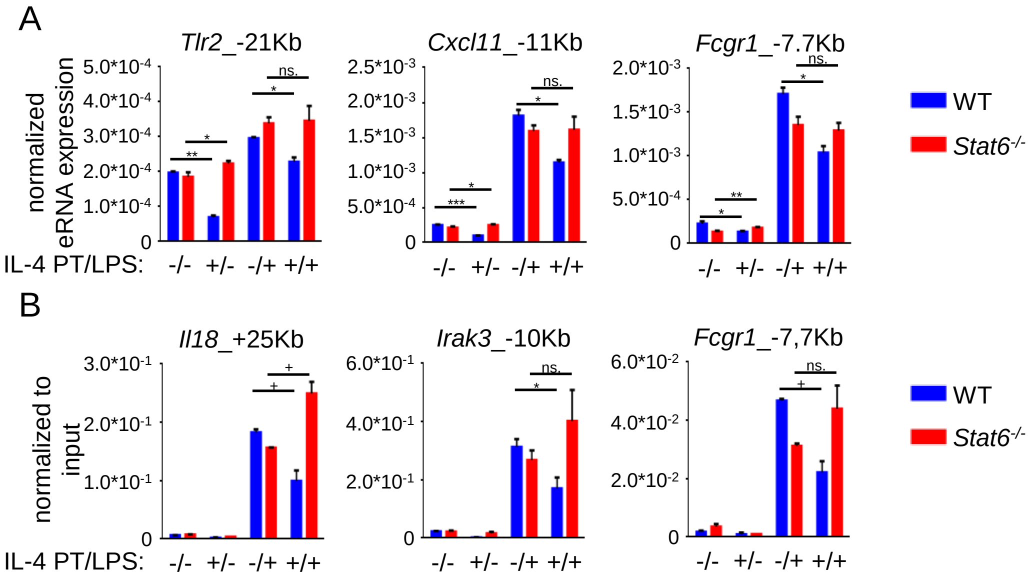

Figure S6. (Related to Figure 6) Investigation of IL-4 pretreatment-madiated repression at the selected LPS-activated enhancers. (A) RT-qPCR-based measurement of basal and LPS-induced eRNA expression at the selected enhancers in IL-4-pre-treated and unstimulated WT and *Stat6*$^{-/-}$ iBMDMs. iBMDMs were pretreated with IL-4 for 24 hours followed by 3 hours LPS exposure. Data represent the mean and SD of three biological replicates. **P*<0.05, ***P*<0.01, ****P*<0.001, ns. indicates not significant. (B) ChIP-qPCR-based measurement of p65 binding at the selected IL-4-repressed LPS-responsive enhancers from WT and *Stat6*$^{-/-}$ BMDMs. BMDMs were pretreated with IL-4 for 24 hours followed by 1 hour LPS exposure. Data represent the mean and SD of two biological replicates. $^{+}$*P*<0.1, **P*<0.5, ns. indicates not significant.

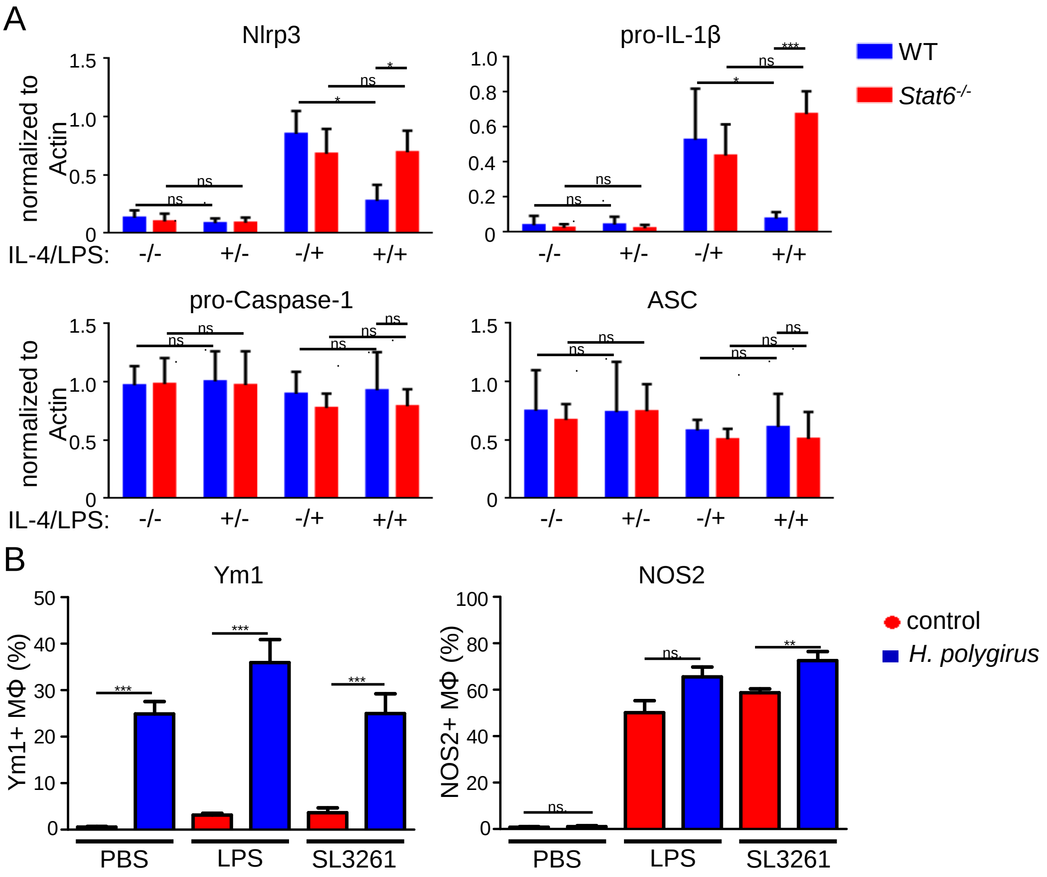

Figure S7. (Related to Figure 7) (A) Western blot densitometry analysis of basal and LPS-regulated Nlrp3, pro-IL-1β, pro-Caspase1, ASC and β-actin expression at protein levels in IL-4-pre-treated and unstimulated WT and *Stat6*$^{-/-}$ mouse BMDMs. BMDMs were pretreated with IL-4 for 24 hours followed by 3 hours LPS exposure. Data represent the mean and SD of five individual animals from two independent experiments. **P*<0.05, ***P*<0.01, ****P*<0.001, ns. indicates not significant. (B) The percentage of Ym1 positive and NOS2 positive peritoneal macrophage number in control and *Heligmosomoides polygyrus* (H. polygyrus)-infected mice following PBS and LPS injection as well as *Salmonella Typhimurium* (SL3261)-infection. Data represent the mean and SD of four-eleven individual animals. **P*<0.05, ***P*<0.01, ****P*<0.001, ns. indicates not significant.
